# Internal OH^−^ induced cascade quenching of upconversion luminescence in NaYF_4_:Yb/Er nanocrystals

**DOI:** 10.1038/s41377-021-00550-5

**Published:** 2021-05-19

**Authors:** Yansong Feng, Zhi Li, Qiqing Li, Jun Yuan, Langping Tu, Lixin Ning, Hong Zhang

**Affiliations:** 1 van’t Hoff Institute for Molecular Sciences, University of Amsterdam, Science Park 904, 1098XH Amsterdam, The Netherlands; 2 State Key Laboratory of Explosion Science and Technology, School of Mechatronical Engineering, Beijing Institute of Technology, Beijing, 100081 China; 3 Anhui Key Laboratory of Optoelectronic Materials Science and Technology, Department of Physics, Anhui Normal University, 241000 Wuhu, China; 4 State Key Laboratory of Luminescence and Applications, Changchun Institute of Optics, Fine Mechanics and Physics, Chinese Academy of Sciences, Changchun, 130033 Jilin China

**Keywords:** Nanophotonics and plasmonics, Nonlinear optics, Nanoparticles

## Abstract

Internal hydroxyl impurity is known as one of the main detrimental factors affecting the upconversion (UC) efficiency of upconversion luminescence (UCL) nanomaterials. Different from surface/ligand-related emission quenching which can be effectively diminished by, e.g., core/shell structure, internal hydroxyl is easy to be introduced in synthesis but difficult to be quantified and controlled. Therefore, it becomes an obstacle to fully understand the relevant UC mechanism and improve UC efficiency of nanomaterials. Here we report a progress in quantifying and large-range adjustment of the internal hydroxyl impurity in NaYF_4_ nanocrystals. By combining the spectroscopy study and model simulation, we have quantitatively unraveled the microscopic interactions underlying UCL quenching between internal hydroxyl and the sensitizers and activators, respectively. Furthermore, the internal hydroxyl-involved UC dynamical process is interpreted with a vivid concept of “Survivor effect,” i.e., the shorter the migration path of an excited state, the larger the possibility of its surviving from hydroxyl-induced quenching. Apart from the consistent experimental results, this concept can be further evidenced by Monte Carlo simulation, which monitors the variation of energy migration step distribution before and after the hydroxyl introduction. The new quantitative insights shall promote the construction of highly efficient UC materials.

## Abstract (web summary)

Exponential relationship between upconversion luminescence intensity and internal OH^-^ content obtained through the combination of quantitative control of the defect, advanced spectroscopy and theoretical model simulation.

## Graphical abstract

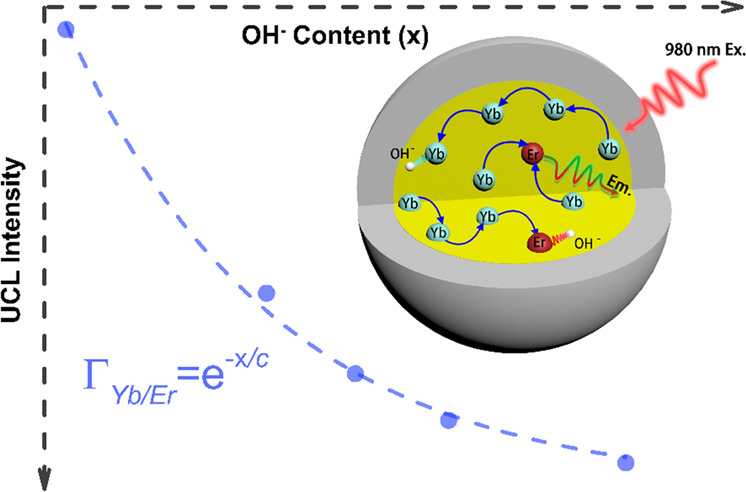

## Introduction

The great application prospect in biology, medicine, optogenetics, photovoltaics, and sustainability has enabled lanthanide (Ln) ion-doped upconversion nanoparticles (UCNPs) to attract widespread attention, which derives mainly from their superior anti-Stokes spectroscopic property^1–13^. Despite a significant progress in synthesis chemistry of UCNPs, the relatively low upconversion (UC) efficiency, especially under restricted excitation power density, e.g., that allowed in clinics, remains a major bottleneck on their way of actual applications^14–18^. Over the past decade, various approaches have been devoted to improve UC efficiency, including tailoring local crystal field, plasmon-enhancement, active Ln^3+^ high-level doping, inorganic–organic hybridization, and defects/impurities deactivation, etc., or to improve the absorption of the near-infrared (NIR) laser with organic dye sensitization^19–31^. Nevertheless, UC efficiency of most reported UCNPs is still significantly far inferior to their bulk counterparts. Admittedly, nanomaterials are much more vulnerable to charged impurities, among which OH^−^ is the most critical, as it may be readily brought into the nanocrystals during synthesis and passivation may increase the UC efficiency by 10–1000 times^32,33^. It is well recognized that there exist in general two kinds of OH^−^: those in ligands or solvents responsible for “surface-related quenching” and those inside the crystal lattice inducing “internal quenching” of UC luminescence (UCL). The quenching mechanism of the surface OH^−^ has been well documented either from the comparison of bare core and core@shell structures or from OH^−^ content variation in solvents^33–39^. The internal OH^−^ impurities are, however, difficult to study because of the obstacle in quantifying the content and relevant reports are thus very limited. On one hand, UC efficiency can be lifted by more than one order of magnitude when measures are carefully taken to eliminate all potential OH^−^ groups in the reactants^40^. On the other hand, the relevant mechanism remains vague. In previous studies, even the actual existence of internal OH^−^, as an important basis for mechanism discussion, usually relies on assumptions^32,40^. Without a quantitative correlation between OH^−^ content and the corresponding UC properties, the comprehension of quenching mechanism would be restricted to a superficial level.

To overcome the long-lasting difficulty, the selection of appropriate methods in quantifying the internal OH^−^ content and analyzing its quantitative relationship with UCL quenching come into an urgent priority. Traditional treatment of UC mechanism based on simultaneous rate equations seems inappropriate for addressing properly the microscopic Ln^3+^-OH^−^ interactions, because it only offers macroscopically averaged statistics. Therefore, it is of particular importance to employ proper theoretical approaches that can not only explain the OH^−^-related steady-state UC phenomena but also provide microscopic details of Ln^3+^-OH^−^/Ln^3+^ interaction, such as the quenching strength of a single OH^−^ to its neighboring excited Ln^3+^ and how this negative effect is bridged to other ions through Ln^3+^–Ln^3+^ interactions.

**Scheme 1:**
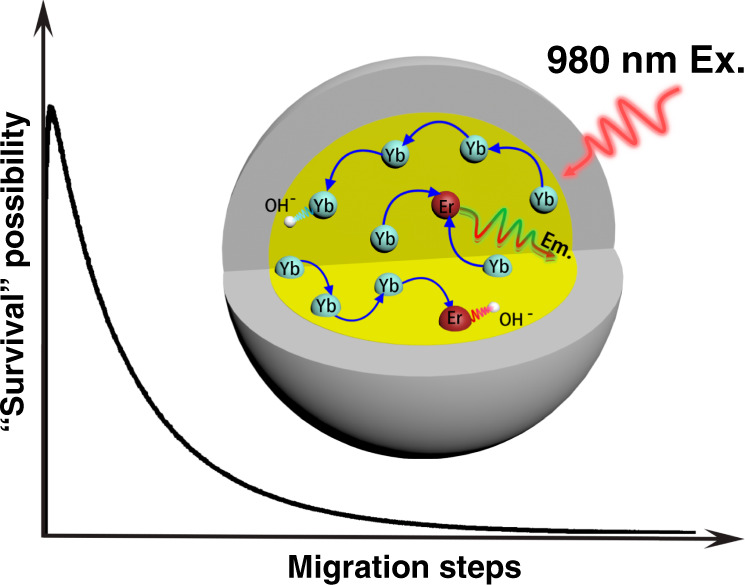
Schematic diagram of the internal OH^−^ influences on energy migration-induced UCL, which can be visualized as “survivor effect.” Typically, the shorter the migration path of an excited state of Yb^3+^, the larger the possibility of its surviving from OH^−^-induced quenching

In this work, we have modified existing protocols and developed a target-oriented dry-control protocol for synthesizing ultra-small core-shell UCNPs to systematically modulate the content of internal OH^−^. It is found that internal OH^−^ content in NaYF_4-*x*_(OH)_*x*_:Yb,Er@NaYF_4_ nanoparticles could be well determined in D_2_O solution from Fourier-transform infrared spectroscopy (FTIR) technique (*x*-value varies from 0 to 0.120). The microscopic quenching mechanism is simulated using Monte Carlo method based on Ln^3+^–Ln^3+^/OH^−^ one-to-one interactions, in which the effect of OH^−^ quenching is explicitly distinguished as activator quenching and sensitizer quenching. Specially, when an OH^−^ locates nearby, the de-excitation rate of Yb^3+^ excited state (^2^F_5/2_ → ^2^F_7/2_) is promoted to about 2100 s^−1^, almost twice faster than that without OH^−^ (735 s^−1^, determined from the experimental data of OH^−^-free sample). On the basis of this variation, the internal OH^−^-induced steady-state UCL quenching of ~30-folds and “accelerated” UC dynamic process (i.e., the shortened rise and decay) are also well simulated. Furthermore, according to the simulation results, we proposed a so-called “survivor effect” to intuitively explain the internal OH^−^ effects on UC, i.e., as shown in Scheme 1, the excited states involved in UC are the survivors of energy migration processes. The shorter the migration path of a photo-excited state of Yb^3+^, the greater its chance of survival and the greater its contribution to UC. The significance of this work is that it provides a clearer insight of impurity-to-ion microscopic interactions in Ln UC processes, which shall pave the way for the pursuit of new structures and/or doping patterns of highly effective UC materials in the future.

## Results

**Fig. 1:**
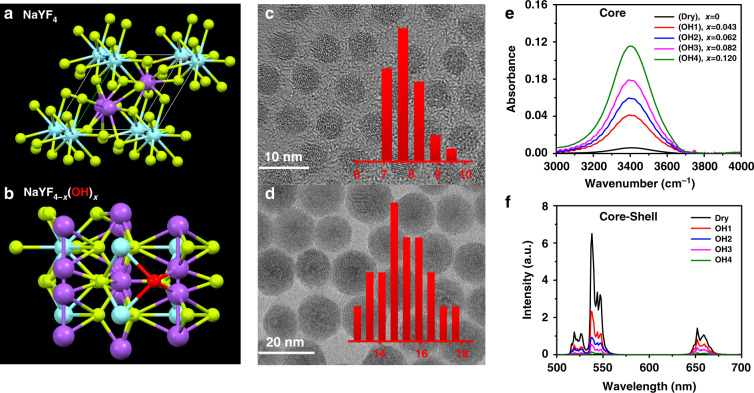
Schematic illustration and characterization of OH^−^ involved UCNPs. Schematic illustration of **a** NaYF_4_ and **b** NaYF_4-*x*_(OH)_*x*_ structures in a unit cell with OH^−^ marked as red ball, Na^+^ as blue ball, Y^3+^ as purple ball, and F^−^ as yellow ball. Typical TEM images of **c** dry core (NaYF_4_:Yb,Er) and **d** dry core-shell (NaYF_4_:Yb,Er@NaYF_4_) UCNPs. Inset: the diameter distribution of the synthesized particles. **e** FTIR spectra of core UCNPs (NaYF_4-*x*_(OH)_*x*_:Yb,Er) containing different amounts of OH^−^ (*x* varies from 0 to 0.120). **f** UCL spectra of the corresponding core-shell UCNPs (NaYF_4-*x*_(OH)_*x*_:Yb,Er@NaYF_4_) in *n*-hexane with 980 nm laser excitation under power density of 10 W cm^−2^

It has been reported that, without strictly drying control in synthesis, OH^−^ contained in the reactants (RECl_3_·6H_2_O and NaOH) or solvents readily incorporate into the crystal lattice^40^. Due to the identical electronic structure of the outer layer, electric charge, and chemical similarity, OH^−^ is likely to substitute F^−^ in the NaYF_4_ sub-lattices, as shown in Fig. 1a, b. As OH^−^-free UCNPs are the premise of the quantitative analysis of internal OH^−^, we have modified the reported protocol^40^ and have developed a convenient procedure to synthesize the ultra-small OH^−^-free UCNPs (Dry core and Dry core-shell structure). The synthesis system was dried with acetic anhydride and the reaction was conducted all-in-one step under nitrogen atmosphere to avoid introduction of H_2_O/OH^−^. To adjust the internal OH^−^ content in core UCNPs (series samples OH1–4), OH^−^ containing reactants (RECl_3_·6H_2_O or NaOH) or watery solvents were employed. As shown in Fig. 1c, d and Supplementary Fig. S1 (high-resolution transmission electron microscopy (TEM)), the diameters of bare core and core-shell UCNPs are 7.5 and 15 nm, respectively, and all UCNPs exhibit pure hexagonal phase (Supplementary Fig. S2) and uniform morphology regardless of their difference in OH^−^ content (Supplementary Figs. S3 and S4). The test results of UC quantum yield (QY) exhibit the importance of removing OH^−^. The QY of Dry core-shell UCNPs reaches 1.3 ± 0.2%, much higher than the OH1–4 series samples (under the excitation of 5 W cm^−2^ 980 nm diode laser, Supplementary Table S1). More importantly, for the first time, through comparing with standard NaOH/D_2_O solution, we made it possible to quantify the OH^−^ content inside the UC nanoparticles with FTIR technique, where ultra-small nanoparticles were employed to guarantee the reliability of quantification (experimental details are depicted in Supplementary Information, the part of “Determination of internal OH^−^ contents in UCNPs”)^39,41^. As displayed in Fig. 1e, the series of core NaYF_4-*x*_(OH)_*x*_-based nanoparticles were labeled as Dry and OH1–4, corresponding to OH^−^ content index *x* of 0, 0.043 ± 0.004, 0.062 ± 0.008, 0.082 ± 0.006, and 0.120 ± 0.008, respectively (Supplementary Table S2). Finally, a thick, dry NaYF_4_ shell was coated to minimize surface effects. It should be noticed that, in this work, the OH^−^ contents were treated essentially unchanged before and after the dry shell coating (the reasons are discussed in detail in the part of “Determination of internal OH^−^ contents in UCNPs” in Supplementary Information and Table S3). After all these measures, the internal OH^−^-dependent UCL of the core-shell structures was obtained, as shown in Fig. 1f.

From a microscopic point of view, UCL can be simplified as the result of a “collision” of two randomly wandering excited states on an activator site^42^. Internal OH^−^ brings in an additional non-radiative process to its neighboring excited Yb^3+^ (sensitizer) or Er^3+^ (activator). As UCL quenching in the co-doping system is induced by Yb^3+^-OH^−^ and Er^3+^-OH^−^ interactions independently, the total quenching effect can be treated as the product of two parts (the detailed modeling process is discussed in the part of “Modelling the simulation” of Supplementary Information), that is:

1 $${{I}}_{{\mathrm{OH}}}{{/I}} = {\Gamma}_{{\mathrm{Er}}} \ast \left( {{\Gamma}_{\mathrm{Yb}}} \right)^2$$

where *I*_OH_/*I* is the ratio of the UCL emission intensities with/without OH^−^, and Γ_Er_ and Γ_Yb_^2^ are the quenching factors of OH^−^ to Er^3+^ and Yb^3+^, respectively. The square form of the Γ_Yb_ represents the nonlinear feature of UCL (in the case of two-photon process).

**Fig. 2:**
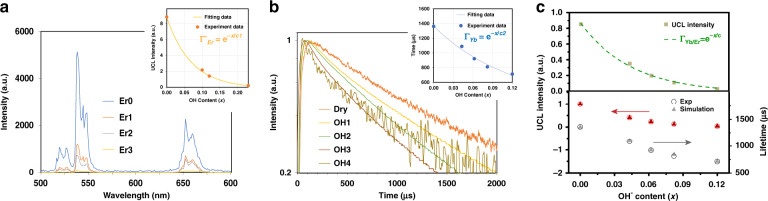
Linear and nonlnear luminescence and relevant simulation results. **a** UCL spectra of NaYF_4-*x*_(OH)_*x*_:2%Er@NaYF_4_ nanoparticles with variable OH^−^ contents in the core (*x*: 0–0.23). Inset shows the corresponding UCL emission intensity (integrated from 500 to 700 nm), which can be well fitted by Eq. (2). **b** Luminescence decay curves of the ^2^F_5/2_ → ^2^F_7/2_ transition of Yb^3+^ in co-doped NaYF_4-*x*_(OH)_*x*_:Yb/Er@NaYF_4_ nanoparticles (Ex: 980 nm, Em: 1020 nm). Inset shows internal OH^−^ content-dependent decay lifetime, which can be well fitted by Eq. (3). **c** Top: internal OH^−^ content-dependent UCL intensity (integrated from 500 to 700 nm) of NaYF_4-*x*_(OH)_*x*_:Yb/Er@NaYF_4_ nanoparticles. Green dots are the experimental values and the curve is the calculated result from Eq. (4); bottom: comparison of experimental (circle) and simulation (triangle, R_Yb-OH_ = 2100 s^−1^) results for the Yb^3+^ decay lifetimes (black color) and UCL intensities (red color) of NaYF_4-*x*_(OH)_*x*_:Yb/Er@NaYF_4_ core/shell nanoparticles

It is, however, difficult to compare directly Eq. (1) with experimental data. To establish an analytical expression to connect the experimental results of Er dopant and Er, Yb co-dopant systems, we have adopted an approximation by replacing one Yb → Er energy transfer process with Er → Er energy transfer process and then Eq. (1) can be approximated as: *I*_OH_/*I* = Γ^’^_Er_ × Γ_Yb_. The new quenching factor Γ^’^_Er_ could then be experimentally obtained from the single Er^3+^-doped NaYF_4-*x*_(OH)_*x*_:2%Er@NaYF_4_ model (Er0–3; the *x*-values for series samples are 0, 0.101 ± 0.008, 0.119 ± 0.013, and 0.230 ± 0.025, respectively), where the UCL is mainly caused by Er → Er sensitization processes^27,43,44^. From Fig. 2a, it is quite gratifying to notice that the UCL profiles can be analyzed with the exponential decay fitting (under 980 nm excitation), which is well in line with other energy quenchers (e.g., surface OH^−^, H_2_O)^35^. Therefore, the factor Γ_Er_^’^ can be expressed as:

2 $${\Gamma}_{{\mathrm{Er}}}^\prime = {{e}}^{ - x/{{c}}_{\mathrm{1}}}$$

where *x* is the OH^−^ content in the nanoparticle and constant *c*_1_ was determined to be 0.072 from the fitting (Fig. 2a inset).

Concerning the parameter Γ_Yb_, it is difficult to get its value directly from Yb^3+^ steady-state emission spectrum, as the emission and excitation spectra of Yb^3+^ overlap heavily. As an alternative, we turned to the time behavior of its luminescence. Obviously, because of the strong energy migration between Yb^3+^ ions (i.e., Yb^3+^ → Yb^3+^), the OH^−^ quenching on its neighboring excited Yb^3+^ ions will diffuse to other Yb^3+^ ions in distance. As predicted by Burshtein^45^, energy migration between Yb^3+^ ions follows the “energy hopping” model. Strict analytical expression of this model in most instances is infeasible, as part of relevant parameters are not experimentally available (details are discussed in Supplementary Information, the part of “Simulation model”). In the current scenario, however, it could be determined experimentally, because the effect of OH^−^ on Yb^3+^ emission decay demonstrates phenomenologically an exponential relation (Fig. 2b inset). Considering the linear relationship between the decay lifetime and emission intensity of Yb^3+^, we can obtain:

3 $${\Gamma}_{{\mathrm{Yb}}} = {{e}}^{ - x/c_2}$$

where *x* is the OH^−^ content in the nanoparticle and constant *c*_2_ was determined to be 0.119 from the fitting (Fig. 2b inset). The exponential dependence could be reasonably understood due to the huge difference between the two interaction rates (according to our simulation, Yb^3+^–Yb^3+^ energy migration rate is almost 50 times larger than Yb^3+^-OH^−^ quenching rate, as shown in Supplementary Table S4). In that case, Yb^3+^ at different positions are connected through the efficient Yb^3+^–Yb^3+^ interaction and OH^−^ quenching effect is therefore not restricted to a single Yb^3+^ anymore.

From Eqs. (1)–(3), the total quenching effect of OH^−^ on UC steady-state emission for the NaYF_4-*x*_(OH)_*x*_:Yb,Er@NaYF_4_ nanoparticles is:

4 $${\Gamma}_{{\mathrm{Yb/Er}}} = {\Gamma}_{\mathrm{Er}} \ast \left( {{\Gamma}_{\mathrm{Yb}}} \right)^2 = {\Gamma}_{\mathrm{Er}}^\prime \ast {\Gamma}_{\mathrm{Yb}} = e^{ - x/c}$$

where *c* equals 8 0.045 as calculated from *c*_1_ and *c*_2_. This picture is validated by the excellent match between prediction of Eq. (4) and the experimental results (Fig. 2c top). Notably, calculated from Eqs. (3) and (4), the OH^−^-induced UCL quenching effects on Er^3+^ (i.e., Γ_Er_) and Yb^3+^ (i.e., Γ_Yb_^2^) can be separately evaluated. Further, the latter one takes the dominant role (~75% ratio), which can be well understood from the relatively high doping concentration of Yb^3+^ in the co-doping UCNPs^35^. More importantly, on the basis of these newly obtained experimental data, it is now possible to quantitatively explore the microscopic Yb^3+^-OH^−^ interaction strength from model simulation. In this case, OH^−^ groups are randomly distributed inside the core and the de-excitation rate of an excited Yb^3+^ with one OH^−^ group in the vicinity is introduced in the simulation model as a new parameter (*R*_Yb-OH_), whereas the rest of the parameters are obtained from our previous work (Supplementary Table S4)^46^. Varying *R*_Yb-OH_ value from 1000 to 5000 s^−1^, the simulation draws the following conclusions after comparing with the experimental data: (1) the exponential relationship between Yb^3+^ excited state lifetime and OH^−^ content (described by Eq. (3)) is validated, as shown in Supplementary Fig S5a, and (2) in the NaYF_4-*x*_(OH)_*x*_Yb,Er@NaYF_4_ UC system, the microscopic parameter *R*_Yb-OH_ is determined to be 2100 s^−1^, which is almost three times as fast as the de-excitation rate of Yb^3+^ without OH^−^ (735 s^−1^, calculated from the experimental results in Supplementary Fig. S11). With this value, the Yb^3+^ decay lifetime and UCL intensity can be well predicted (Fig. 2c lower part and Supplementary Fig. S5b).

**Fig. 3:**
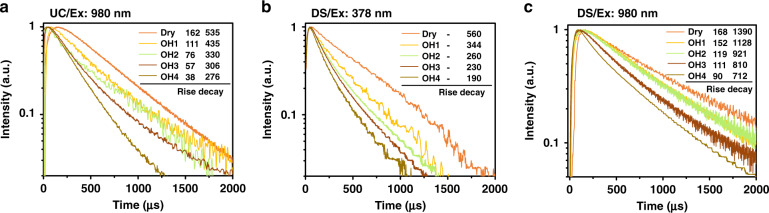
OH^−^ content dependent UC and DS luminescence dynamical curves. **a** OH^−^ content dependence of UC green emission dynamics of co-doped core-shell UCNPs (NaYF_4-*x*_(OH)_*x*_:Yb,Er@NaYF_4_) monitored at ~540 nm. **b** The DS green emission dynamics of core-shell UCNPs (NaYF_4-*x*_(OH)_*x*_:Yb,Er@NaYF_4_) with different OH^−^ contents (excitation: 378 nm and emission: 540 nm). **c** The DS NIR emission dynamics of core-shell UCNPs (NaYF_4-*x*_(OH)_*x*_:Yb,Er@NaYF_4_) with different OH^−^ contents (Ex: 980 nm and Em: 1530 nm). Numbers marked in Fig. 3 are the rise time and decay lifetimes of the series curves (unit: μs)

Next, we discuss the OH^−^ quenching effect on UC dynamical process. As expected, the existence of internal OH^−^ “accelerates” the UC process. From Fig. 3a, the rise time constant of UCL trace decreases for the green (^4^S_3/2_ → ^4^I_15/2_, 540 nm, from 162 to 38 μs) and red emission (Fig S64, F_9/2_ → ^4^I_15/2_, 654 nm, from 270 to 20 μs) upon the OH^−^ content increase. Similar trends were observed for the decay lifetime, which decreases from 535 to 276 μs for the green emission and from 629 to 252 μs for the red emission (Fig. 3a and Supplementary Fig. S6). These changes come partly from the Er^3+^-OH^−^ interaction, which increases the non-radiative relaxation rates of relevant emitting levels of Er^3+^. The solid evidence is provided by the linear down-shifting (DS) emission (Ex: 378 nm, Em: 540 nm) where only the activator Er^3+^, rather than the sensitizer Yb^3+^, could be activated. The decay lifetime of the green emission decreases significantly from 560 to 190 μs by OH^−^, whereas the rise remains unchanged in the meantime (Fig. 3b). On the other hand, the shortening of the rise part of UCL reappears in the DS NIR emission under 980 nm excitation (Fig. 3c, Em: 1530 nm, decreases from 168 to 90 μs). This recurrence indicates a common process in Fig. 3a, c: OH^−^ involved energy migration among Yb^3+^ ions.

**Fig. 4:**
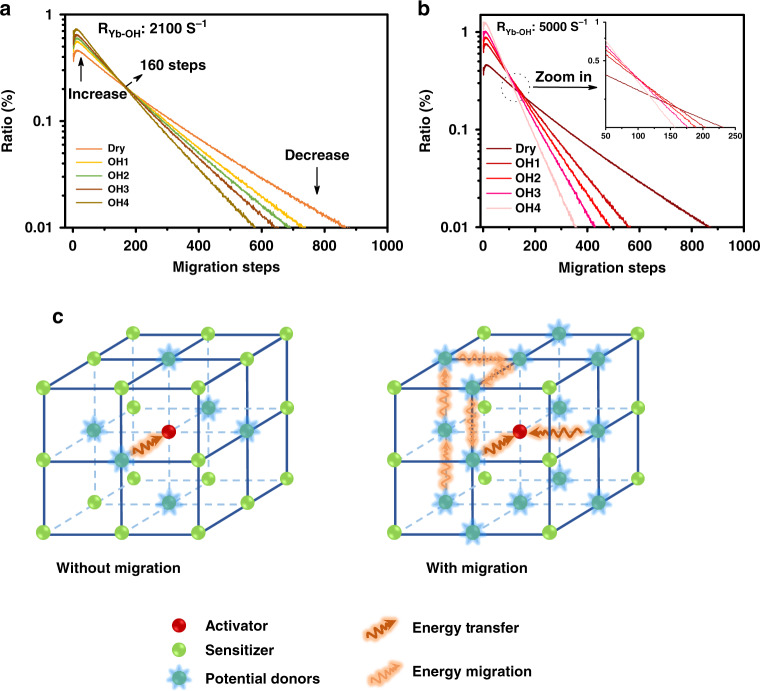
Simulated Yb^3+^–Yb^3+^ energy migration step distribution. The simulated Yb^3+^–Yb^3+^ migration steps distribution in series OH^−^ influenced samples (Dry, OH1–4 core/shell structures): **a** the *R*_Yb-OH_ is set to 2100 s^−1^, **b** the *R*_Yb-OH_ is set to 5000 s^−1^. Each curve in **a**, **b** is normalized by its area. **c** Schematic diagram of the migration steps related Yb^3+^ → Er^3+^ energy transfer processes

Further, Monte Carlo simulation was applied to collect more specific information hidden behind the luminescence variation. As shown in Fig. 4a, b, we counted the Yb^3+^–Yb^3+^ migration step distribution in the OH^−^ involved UC dynamical processes (every curve in Fig. 4a, b is normalized by its area, the migration steps start counting from the initial absorption and end with the excited states reaching to the UC emission energy level of Er^3+^). These simulations have brought us two detailed results, which are difficult to be acquired directly from experiments: (1) as shown in Fig. 4a, with the growing of internal OH^−^ content, the averaged migration steps will be reduced. In detail, the probability of relatively short-ranged migration (<160 steps) increases, whereas the long-ranged migration (>160 steps) probability decreases correspondingly. This phenomenon can be ascribed to the mechanism, which is named here as a “survivor effect,” i.e., obviously, the excited states involved in UC are the survivors of energy migration processes, where energy will be continually dissipated via radiative or non-radiative relaxation. Therefore, the shorter the migration path experienced by an excited state, the greater the chance of its survival and the greater the chance of its participating in UC. Moreover, “survivor effect” will become more remarkable with additional energy quenchers introduced, where internal OH^−^ is a typical example. By impeding the long-ranged migration more seriously, internal OH^−^ promotes the relative contribution share of short-ranged migration in UCL and UC dynamical process will be “accelerated” by saving the averaged energy migration steps (appearing as the shortening of the rise and decay of UCL in Fig. 3a). Simulation shows further that the cross-point of these curves (i.e., at 160 steps in Fig. 4a, which also means the boundary to distinguish long- and short-ranged energy migration) is not always a fixed value if the quenching rate of the internal defects (i.e., *R*_Yb-defect_) becomes much larger, e.g., 5000 s^−1^ in Fig. 4b (or varied from 10^3^ to 10^4^ s^−1^ in Supplementary Fig. S7). In spite of this, the variation tendency of the long/short-ranged migration remains unchanged. (2) There is a short rise at the beginning of the curves (step 1–10 in Fig. 4a, b). In our opinion, it represents a trade-off between two opposite effects. As shown in Fig. 4c, on one hand, Yb^3+^ ion is randomly excited and it cannot be ensured that there is exactly one Er^3+^ nearby to accept its energy. Considering this point, certain steps of Yb^3+^ → Yb^3+^ migration are of benefit to the efficient Yb^3+^ → Er^3+^ energy transfer by allowing the Er^3+^ to harvest energy from more distant Yb^3+^ ions. On the other hand, the continuous energy loss during the migration process, as a negative factor, will set up the ceiling of the migration steps. As a result, the peaking point is located at the moment of a ten-step migration, where a balance of this trade-off is achieved.

**Fig. 5:**
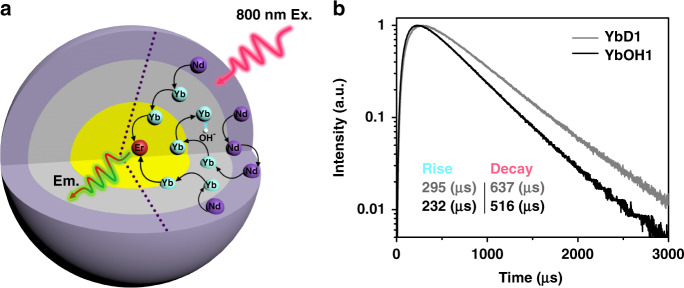
Schematic illustration and UC emission dynamical curves of the Yb,Er@Yb(OH)_*x*_@Nd structure. **a** The UC process in the Yb,Er@Yb(OH)_*x*_@Nd structure, in which the three basic processes (photon absorption (800 nm), energy migration, and UC emission) are spatially separated. Black curve arrows represent the energy transfer steps affected by the OH^−^ quenching. **b** Under 800 nm excitation, the UC emission (540 nm) dynamical processes of different middle layer samples: without OH^−^ (YbD1) and with OH^−^ (YbOH1). The middle layer thicknesses are ca. 2.3 nm for YbD1 and YbOH1

Furthermore, to verify the universality of “survivor effect,” another UC model system (Fig. 5a), i.e., Yb,Er@Yb(OH)_*x*_@Nd was prepared, where the energy donor (sensitizer) and acceptor (activator) are spatially separated (rather than mixed co-doping), and the amount of OH^−^ is varied in the middle layer to affect mainly the sensitizers during the energy migration. As expected, similar phenomena appear to be also originated from the survivor effect, e.g., under the influence of internal OH^−^ quencher, both rise and decay times of UC dynamical processes decrease upon 800 nm excitation, which is ascribed to the shortened energy migration time in the middle layer (2.3 nm), as shown in Fig. 5b. Survivor effect is also validated when the migration layer thickness changes to 3.3 nm or detection for the emission detection changes to 654 nm (Supplementary Fig. S8).

## Discussion

In summary, the effect of OH^−^ defect inside nanocrystals on UC is studied from the views of both theoretical simulation and spectroscopy with specifically designed nanostructures. An all-in-one dry method is developed to synthesize highly efficient ultra-small nanoparticles and manipulate OH^−^ contents in the specific areas of the model nanoparticles. By integrating the OH^−^ manipulating synthesis, spectroscopy, and corresponding Monte Carlo simulation of a series of core/shell and core/shell/shell model samples, we have quantitatively acquired the exponential relation between UCL intensity and the quantity of internal OH^−^. The microscopic quenching picture of OH^−^ on UC is distinctly unraveled with the successful separation of OH^−^-activator and OH^−^-sensitizer interactions. “Survivor effect” is proposed and validated to play a non-negligible role in OH^−^-induced UCL quenching. These results have disentangled part of the long-standing impurity-related puzzles in Ln-based UC materials, which paves the way to our pursuit of new structures and/or doping patterns for higher UC efficiency.

## Materials and methods

### Reagents

RE(CH_3_COO)_3_·*x*H_2_O (RE: Y, Yb, Er, Nd) (99.9% metals basis), RECl_3_·6H_2_O (99.9%), oleic acid (90%), 1-octadecene (ODE, 90%), NaOH, NH_4_F, sodium oleate (NaOA), acetic acid, acetic anhydride, methanol, ethanol, acetone, and cyclohexane were purchased from Sigma-Aldrich. All the chemicals were of analytical grade and were used without further purification.

### Synthesis of dry core β-NaYF_4_:Yb,Er nanoparticles

ODE (10 mL) and 10 mL oleic acid were heated up to 100 °C and kept under vacuum for 60 min. Y(CH_3_COO)_3_·*x*H_2_O (0.78 mmol), 0.20 mmol Yb(CH_3_COO)_3_·*x*H_2_O, and 0.02 mmol Er(CH_3_COO)_3_·*x*H_2_O were then added in under nitrogen flow. Subsequently, 0.5 mL acetic anhydride was injected in the solution under nitrogen and reacted at 100 °C for 60 min, to remove H_2_O/OH^−^ in the system. Acetic anhydride was removed under vacuum at 100 °C. Thereafter, 2.8 mmol NaOA was added under nitrogen flow and switched to vacuum until the reagents were fully dissolved. NH_4_F (5.8 mmol) was then added in under nitrogen flow and the solution was kept at 100 °C under vacuum for 60 min, to assure complete removal of water or other H_2_O/OH^−^ before nanoparticle growth. Finally, the solution was heated up to 300 °C with the rate of 10 °C min^−1^ and reacted for another 60 min under dry nitrogen. After being cooled down to room temperature, the core nanoparticles were obtained through centrifugation and washed with acetone and ethanol (twice), and finally dispersed in 4 mL cyclohexane.

### Synthesis of core β-NaYF_4-*x*_OH_*x*_:Yb,Er nanoparticles with NaOH—OH1

To synthesize β-NaYF_4-*x*_OH_*x*_:Yb,Er nanoparticles, the solvent and reagents were dried in the same way as aforementioned for the dry nanoparticles. In this case, 2.8 mmol NaOH and 5.8 mmol NH_4_F in 5 mL methanol were added dropwise. The solution was heated to 75 °C and kept for 30 min, to remove methanol under nitrogen flow. Finally, the solution was heated up to 300 °C with the rate of 10 °C min^−1^ and reacted for another 60 min. After being cooled down to room temperature, the core nanoparticles OH1 were obtained through centrifugation and washed with acetone and ethanol, and finally dispersed in 4 mL cyclohexane.

### Synthesis of core β-NaYF_4-*x*_OH_*x*_:Yb,Er nanoparticles with RECl_3_·6H_2_O—OH2

ODE (10 mL) and 10 mL oleic acid were heated up to 100 °C and kept under vacuum for 60 min. YCl_3_·6H_2_O (0.78 mmol), 0.20 mmol YbCl_3_·6H_2_O, and 0.02 mmol ErCl_3_·6H_2_O were then added in under nitrogen flow and kept at 100 °C for 60 min until the reagents dissolved. Thereafter, 2.8 mmol NaOA was added under nitrogen flow and switched to vacuum until the reagents fully dissolved. NH_4_F (5.8 mmol) was then added in under nitrogen flow. Finally, the solution was heated up to 300 °C with the rate of 10 °C min^−1^ and reacted for another 60 min under nitrogen. After being cooled down to room temperature, the nanoparticles OH2 were obtained through centrifugation and washed with acetone and ethanol, and finally dispersed in 4 mL cyclohexane.

### Synthesis of core β-NaYF_4-*x*_OH_*x*_:Yb,Er nanoparticles without further dried solvent—OH3

Y(CH_3_COO)_3_·*x*H_2_O (0.78 mmol), 0.20 mmol Yb(CH_3_COO)_3_·*x*H_2_O, and 0.02 mmol Er(CH_3_COO)_3_·*x*H_2_O were added in 10 mL ODE and 10 mL oleic acid, and heated up to 100 °C for 60 min until the reagents dissolved under nitrogen. Thereafter, 2.8 mmol NaOA was added in under nitrogen flow and switched to vacuum until the reagents fully dissolved. NH_4_F (5.8 mmol) was then added in under nitrogen flow. Finally, the solution was heated up to 300 °C with the rate of 10 °C min^−1^ and reacted for another 60 min. After being cooled down to room temperature, the nanoparticles OH3 were obtained through centrifugation and washed with acetone and ethanol, and finally dispersed in 4 mL cyclohexane.

### Synthesis of core β-NaYF_4-*x*_OH_*x*_:Yb,Er nanoparticles with NaOH, RECl_3_·6H_2_O, and without further dried solvent—OH4

YCl_3_·6H_2_O (0.78 mmol), 0.20 mmol YbCl_3_·6H_2_O, and 0.02 mmol ErCl_3_·6H_2_O were added in 10 mL ODE and 10 mL oleic acid, and heated up to 100 °C for 60 min until the reagents dissolved under nitrogen. NaOH (2.8 mmol) and 5.8 mmol NH_4_F in 5 mL methanol were then added in dropwise. The solution was heated to 75 °C and kept for 30 min to remove methanol under nitrogen flow. Finally, the solution was heated up to 300 °C with the rate of 10 °C min^−1^ and reacted for another 60 min. After being cooled down to room temperature, the nanoparticles OH4 were obtained through centrifugation and washed with acetone and ethanol, and finally dispersed in 4 mL cyclohexane.

### Synthesis of dry core β-NaYF_4_:2%Er nanoparticles

The synthesis route for dry core β-NaYF_4_:2%Er (Er0) was the same as that for dry core β-NaYF_4_:Yb,Er, except 0.98 mmol Y(CH_3_COO)_3_·*x*H_2_O, and 0.02 mmol Er(CH_3_COO)_3_·*x*H_2_O were used.

The synthesis route for β-NaYF_4-*x*_OH_*x*_:2%Er core nanoparticles (Er1, Er2) was the same as that for OH4 nanoparticles except:

Er1: with NaOH, 0.98 mmol Y(CH_3_COO)_3_·*x*H_2_O, and 0.02 mmol Er(CH_3_COO)_3_·*x*H_2_O.

Er2: with NaOA, 0.98 mmol YCl_3_·6H_2_O and 0.02 mmol ErCl_3_·6H_2_O.

For the Er3 core nanoparticles: 0.98 mmol YCl_3_·6H_2_O and 0.02 mmol ErCl_3_·6H_2_O were added in 10 mL ODE and 10 mL oleic acid, and stirred for 60 min until the reagents dissolved. Then 2.8 mmol NaOH and 5.8 mmol NH_4_F were added in. After all the powders were dissolved, the solution was heated up to 300 °C at a rate of 10 °C min^−1^ and reacted for another 60 min under nitrogen. After being cooled down to room temperature, the nanoparticles Er3 were obtained through centrifugation and washed with acetone and ethanol, and finally dispersed in 4 mL cyclohexane.

### Synthesis of dry α-NaYF_4_ precursor

Oleic acid (160 mL) and 160 mL ODE in 500 mL a three-neck flask were heated up to 100 °C and kept under vacuum for 60 min, 20 mmol Y(CH_3_COO)_3_·*x*H_2_O was then added in under nitrogen flow, and, subsequently, 5 mL acetic anhydride was injected in the solution under nitrogen and heated up to 100 °C for 60 min. Acetic anhydride was removed under vacuum at 100 °C. Thereafter, 30 mmol NaOA was added in under nitrogen flow and switched to vacuum until the reagents fully dissolved. NH_4_F (80 mmol) was then added in under nitrogen flow and the solution was heated up to 100 °C under vacuum for 60 min to completely remove water or other H_2_O/OH^−^ sources before nanoparticles growth. Finally, the solution was heated up to 200 °C with a rate of 10 °C min^−1^ and reacted for another 60 min under dry nitrogen. After being cooled down to room temperature, the precursor was obtained through centrifugation and finally dispersed in 40 mL dry ODE.

### Synthesis of dry α-NaYF_4_:Yb precursor and α-NaYF_4-*x*_OH_*x*_:Yb

The synthesis route for dry α-NaYF_4_:Yb was the same as that for dry α-NaYF_4_, except that 16 mmol Y(CH_3_COO)_3_·*x*H_2_O and 4 mmol Yb(CH_3_COO)_3_·*x*H_2_O was used To synthesize α-NaYF_4-*x*_OH_*x*_:Yb, 16 mmol YCl_3_·6H_2_O and 4 mmol YbCl_3_·6H_2_O were added in 160 mL oleic acid and 160 mL ODE, and heated up to 100 °C under nitrogen flow for 60 min until the reagents dissolved. Subsequently, 30 mmol NaOH was dissolved by stirring at 100 °C and 80 mmol NH_4_F was then added in. Finally, the solution was heated up to 200 °C and reacted for 60 min. After being cooled down to room temperature, the precursor was obtained by centrifugation and dispersed in 40 mL ODE.

### Synthesis of dry α-NaYF_4_:Nd precursor

The synthesis route for dry α-NaYF_4_:Nd was the same as that of dry α-NaYF_4_, except that 16 mmol Y(CH_3_COO)_3_·*x*H_2_O and 4 mmol Nd(CH_3_COO)_3_·*x*H_2_O was used.

### Synthesis of β-NaYF_4-*x*_OH_*x*_:Yb,Er@NaYF_4_ core/shell nanoparticles

Core β-NaYF_4-*x*_OH_*x*_:Yb,Er (0.5 mmol; Dry, OH1, OH2, OH3 or OH4) nanoparticles and 2.5 mmol α-NaYF_4_ in 10 mL ODE and 10 mL oleic acid were heated up to 100 °C and kept under vacuum for 60 min. The solution was then heated up to 300 °C and reacted for 90 min. After cooling down to room temperature, the core/shell nanoparticles (Dry, OH1, OH2, OH3, or OH4 NPs) were obtained by centrifugation and washed with acetone and ethanol.

### Synthesis of β-NaYF_4-*x*_OH_*x*_:2%Er@NaYF_4_ core/shell nanoparticles

The synthesis route was the same as that of β-NaYF_4-*x*_OH_*x*_:Yb,Er@NaYF_4_ core/shell nanoparticles, except that core β-NaYF_4-*x*_OH_*x*_:2%Er (Er0, Er1, Er2, and Er3) were used.

### Synthesis of β-NaYF_4_:Yb,Er@NaYF_4_:Yb@NaYF_4_:Nd (1 : 3 : 5 or 1 : 5 : 6.4) core/shell nanoparticles

Dry core β-NaYF_4_:Yb,Er (0.5 mmol; Dry) nanoparticles and 1.5 or 2.5 mmol α-NaYF_4_:Yb in 10 mL ODE and 10 mL oleic acid were heated up to 100 °C and kept under vacuum for 60 min. The solution was quickly heated up to 300 °C and reacted for 30 min, then 2.5 mmol or 3.2 mmol α-NaYF_4_:Nd was injected in and reacted for another 30 min. After cooling down to room temperature, the core/shell nanoparticles β-NaYF_4_:Yb, Er@NaYF_4_:Yb@NaYF_4_:Nd (1 : 3 : 5 or 1 : 5 : 6.4) were obtained by centrifugation and washed with acetone and ethanol.

### Synthesis of β-NaYF_4_:Yb,Er@NaYF_4-*x*_OH_*x*_:Yb@NaYF_4_:Nd (1 : 3 : 5 or 1 : 5 : 6.4) core/shell nanoparticles

The synthesis route was the same as that given above, except that α-NaYF_4-*x*_OH_*x*_:Yb was used here.

### Determination of OH^−^ contents in bare core UCNPs

UCNPs were stirred in 0.1 M DCl (in D_2_O solvent) to remove all the potential OH^−^ in surface ligands. Then the nanoparticles were centrifuged and re-dispersed in pure D_2_O twice and finally dispersed in D_2_O and adjusted to 50 mg mL^−1^. To determine the internal OH^−^ contents, the UCNP solutions (in D_2_O) were separated in triplicate, the content of OH^−^ were determined from its absorbance near 3400 cm^−1^, and measured with FTIR spectroscopy for three times (taking NaOH/D_2_O solution as a standard). A detailed example was given in Supplementary Information.

### Characterization

FTIR absorption was measured using a Bruker Vertex 70 spectrometer in combination with a PMA 50 module for polarization modulation measurements. The center frequency of photoelastic modulator was set to 1400 cm^−1^. A CaF_2_ transmission cell with a path length of 50 μm was used. TEM and X-ray powder diffraction were used to determine the mean size, the size distribution, as well as the crystal phase and the phase purity of all nanoparticles, respectively. UCL QY was measured on the setup described in previous report^47^.

### Theoretical calculations

The steady-state and time evolution of UCL were simulated by a Monte Carlo model, which was upgraded from our previous work (adding the internal OH^−^ quenching as a new parameter)^46^. In this model, a nanoparticle is treated as a three-dimensional sub-lattice (consisted by sensitizer and activator ions). By setting the proper parameters (listed in Supplementary Table S4), we were able to trace the motion tracks of each excited state in the system. The macroscopic phenomena (i.e., absorption and UC emission) were rebuilt by the statistical results of the numerous ion-to-ion interaction processes. More details were given in Supplementary Information.

## Supplementary information

Supplementary Information

## References

[1] Auzel F (2004). Upconversion and anti-stokes processes with f and d ions in solids. Chem. Rev..

[2] Liu GK (2015). Advances in the theoretical understanding of photon upconversion in rare-earth activated nanophosphors. Chem. Soc. Rev..

[3] Tu LP (2015). Excitation energy migration dynamics in upconversion nanomaterials. Chem. Soc. Rev..

[4] Chen GY (2014). Upconversion nanoparticles: design, nanochemistry, and applications in theranostics. Chem. Rev..

[5] Zhou B (2015). Controlling upconversion nanocrystals for emerging applications. Nat. Nanotechnol..

[6] Wang F (2018). Microscopic inspection and tracking of single upconversion nanoparticles in living cells. Light. Sci. Appl..

[7] Zuo J (2018). Near infrared light sensitive ultraviolet-blue nanophotoswitch for imaging-guided “off-on” therapy. ACS Nano.

[8] Yang DM (2013). Hollow structured upconversion luminescent NaYF_4_:Yb^3+^, Er^3+^ nanospheres for cell imaging and targeted anti-cancer drug delivery. Biomaterials.

[9] Hou ZY (2019). Hydrogenated titanium oxide decorated upconversion nanoparticles: facile laser modified synthesis and 808 nm near-infrared light triggered phototherapy. Chem. Mater..

[10] Teh DBL (2020). A flexi-PEGDA upconversion implant for wireless brain photodynamic therapy. Adv. Mater..

[11] Chen S (2018). Near-infrared deep brain stimulation via upconversion nanoparticle – mediated optogenetics. Science.

[12] Liang LL (2019). Upconversion amplification through dielectric superlensing modulation. Nat. Commun..

[13] Ji YN (2020). Huge upconversion luminescence enhancement by a cascade optical field modulation strategy facilitating selective multispectral narrow-band near-infrared photodetection. Light. Sci. Appl..

[14] Boyer JC, van Veggel FCJM (2010). Absolute quantum yield measurements of colloidal NaYF_4_: Er^3+^, Yb^3+^ upconverting nanoparticles. Nanoscale.

[15] Faulkner DO (2012). Absolute quantum yields in NaYF_4_:Er,Yb upconverters - synthesis temperature and power dependence. J. Mater. Chem..

[16] Wang F, Wang J, Liu XG (2010). Direct evidence of a surface quenching effect on size-dependent luminescence of upconversion nanoparticles. Angew. Chem. Int. Ed..

[17] Rinkel T (2016). Synthesis of 10 nm β-NaYF_4_:Yb,Er/NaYF_4_ core/shell upconversion nanocrystals with 5 nm particle cores. Angew. Chem. Int. Ed..

[18] Zhao JB (2013). Upconversion luminescence with tunable lifetime in NaYF_4_:Yb,Er nanocrystals: role of nanocrystal size. Nanoscale.

[19] Chen G (2008). Upconversion emission enhancement in Yb^3+^/Er^3+^-codoped Y_2_O_3_ nanocrystals by tridoping with Li^+^ ions. J. Phys. Chem. C.

[20] He JJ (2017). Plasmonic enhancement and polarization dependence of nonlinear upconversion emissions from single gold nanorod@SiO_2_@CaF_2_:Yb^3+^, Er^3+^ hybrid core–shell–satellite nanostructures. Light. Sci. Appl..

[21] Zhang F (2010). Fabrication of Ag@SiO_2_@Y_2_O_3_:Er nanostructures for bioimaging: tuning of the upconversion fluorescence with silver nanoparticles. J. Am. Chem. Soc..

[22] Zhou JJ (2018). Activation of the surface dark-layer to enhance upconversion in a thermal field. Nat. Photonics.

[23] Zou WQ (2012). Broadband dye-sensitized upconversion of near-infrared light. Nat. Photonics.

[24] Garfield DJ (2018). Enrichment of molecular antenna triplets amplifies upconverting nanoparticle emission. Nat. Photonics.

[25] Xu JT (2017). Highly emissive dye-sensitized upconversion nanostructure for dual-photosensitizer photodynamic therapy and bioimaging. ACS Nano.

[26] Yi GS, Chow GM (2007). Water-soluble NaYF_4_:Yb,Er(Tm)/NaYF_4_/polymer core/shell/shell nanoparticles with significant enhancement of upconversion fluorescence. Chem. Mater..

[27] Johnson NJJ (2017). Direct evidence for coupled surface and concentration quenching dynamics in lanthanide-doped nanocrystals. J. Am. Chem. Soc..

[28] Zuo J (2017). Employing shells to eliminate concentration quenching in photonic upconversion nanostructure. Nanoscale.

[29] Chen X (2016). Confining energy migration in upconversion nanoparticles towards deep ultraviolet lasing. Nat. Commun..

[30] Wen SH (2018). Advances in highly doped upconversion nanoparticles. Nat. Commun..

[31] Liu Q (2018). Single upconversion nanoparticle imaging at sub-10 W cm^-2^ Irradiance. Nat. Photonics.

[32] Rabouw FT (2018). Quenching pathways in NaYF_4_:Er^3+^,Yb^3+^ upconversion nanocrystals. ACS Nano.

[33] Guo SH (2016). Sensitive water probing through nonlinear photon upconversion of lanthanide-doped nanoparticles. ACS Appl. Mater. Interfaces.

[34] Chen DQ (2017). Water detection through Nd^3+^-sensitized photon upconversion in core-shell nanoarchitecture. J. Mater. Chem. C.

[35] Arppe R (2015). Quenching of the upconversion luminescence of NaYF_4_:Yb^3+^,Er^3+^ and NaYF_4_:Yb^3+^,Tm^3+^ nanophosphors by water: the role of the sensitizer Yb^3+^ in non-radiative relaxation. Nanoscale.

[36] Hyppänen I (2017). Environmental impact on the excitation path of the red upconversion emission of nanocrystalline NaYF_4_:Yb^3+^,Er^3+^. J. Phys. Chem. C.

[37] Boyer JC (2010). Surface modification of upconverting NaYF_4_ nanoparticles with PEG-phosphate ligands for NIR (800 nm) biolabeling within the biological window. Langmuir.

[38] Stouwdam JW (2003). Lanthanide-doped nanoparticles with excellent luminescent properties in organic media. Chem. Mater..

[39] De GJH (2006). Effect of OH^−^ on the upconversion luminescent efficiency of Y_2_O_3_:Yb^3+^, Er^3+^ nanostructures. Solid State Commun..

[40] Homann C (2018). NaYF_4_:Yb,Er/NaYF_4_ core/shell nanocrystals with high upconversion luminescence quantum yield. Angew. Chem. Int. Ed..

[41] Baumer A, Ganteaume M, Klee WE (1985). Determination of OH ions in hydroxyfluorapatites by infrared spectroscopy. Bull. Minéral..

[42] Zhang JH (2015). Observation of efficient population of the red-emitting state from the green state by non-multiphonon relaxation in the Er^3+^–Yb^3+^ system. Light. Sci. Appl..

[43] Chen QS (2017). Confining excitation energy in Er^3+^-sensitized upconversion nanocrystals through Tm^3+^-mediated transient energy trapping. Angew. Chem. Int. Ed..

[44] Vetrone F (2003). Concentration-dependent near-infrared to visible upconversion in nanocrystalline and bulk Y_2_O_3_:Er^3+^. Chem. Mater..

[45] Brushteǐn AI (1972). Hopping mechanism of energy transfer. Sov. J. Exp. Theor. Phys..

[46] Zuo J (2018). Precisely tailoring upconversion dynamics via energy migration in core-shell nanostructures. Angew. Chem. Int. Ed..

[47] Meijer MS (2018). Absolute upconversion quantum yields of blue-emitting LiYF_4_:Yb^3+^,Tm^3+^ upconverting nanoparticles. Phys. Chem. Chem. Phys..

