# Supplementary material for: Internal OH− induced cascade quenching of upconversion luminescence in NaYF4:Yb/Er nanocrystals
Source: Light Sci Appl. 2021 May 19;10:105. doi: 10.1038/s41377-021-00550-5 (PMC8134431; doi:10.1038/s41377-021-00550-5)
Supplement: Supplementary file 1 — Supplementary Information [file 41377_2021_550_MOESM1_ESM.docx]

**Supplementary Information for**

**Internal OH^-^ Induced Cascade Quenching of Upconversion Luminescence in NaYF_4_:Yb/Er Nanocrystals**

Yansong Feng,^†a,d^ Zhi Li,^†c^ Qiqing Li,^a^ Jun Yuan,^a^ Langping Tu,^b,^* Lixin Ning,^c,^* Hong Zhang ^a,^*

[a] Van ’t Hoff Institute for Molecular Sciences, University of Amsterdam, Science Park 904, 1098 XH Amsterdam, The Netherlands

[b] State Key Laboratory of Luminescence and Applications Changchun Institute of Optics, Fine Mechanics and Physics, Dong Nan Hu road 3888, 130033 Changchun, China

[c] Anhui Key Laboratory of Optoelectronic Materials Science and Technology, Department of Physics, Anhui Normal University, 241000, Wuhu, China

[d] State Key Laboratory of Explosion Science and Technology, School of Mechatronical Engineering, Beijing Institute of Technology, Beijing 100081, China

**Determination of internal OH^-^ contents in UCNPs**:

Since internal OH^-^ contents determination is the base of all subsequent analyses, a well- designed experimental procedure is essential. The reliability of our results is based on the following reasons:

(1) Despite this is the first report for the quantitative analysis on internal OH^-^ content in UCNPs via Fourier-Transform Infrared spectroscopy (FTIR) technology, the feasibility of this quantitative strategy has been verified previously, where FTIR was utilized to determine the OH^-^ contents inside the Ca_5_(PO_4_)_3_(OH)*_x_*F_1-_*_x_* bulk crystal by referring to the absorption intensity of OH^-^ vibrations.^S1^

(2) In order to collect reliable data of bare core structure, we used a high concentration of UCNPs dispersed in pure D_2_O (50 mg mL^-1^, the ultra-small particle size guarantees the high dispersed concentration in D_2_O without precipitation) to secure a high signal to noise, which can reduce the error.

(3) We are able to manifest the reliability of our strategy by observing almost zero-intensity of OH^-^ vibrations in absolutely dried UCNPs, but obvious OH^-^ vibration peaks in samples with only artificially added OH^-^ on purpose (as shown in **Figure 1e** and **Figure** **S9**). And the error bar of our measured results is less than 15% (**Table S2**), which is acceptable in this work.

Here, we take β-NaREF_4-_*_x_*(OH)*_x_* bare core structure as an example. To exclude the influence of surface OH^-^ group, we used 0.1 M DCl (in D_2_O) instead of HCl (in H_2_O) to remove all the potential OH^-^ in surface ligands/H_2_O molecules. Briefly, 4 mL β-NaREF_4-_*_x_*OH*_x_* core nanoparticles and 4 mL 0.1 M DCl were mixed and stirred vigorously for 4 h until the nanoparticles were completely transferred into the D_2_O phase. The nanoparticles were centrifuged and re-dispersed in pure D_2_O twice and finally dispersed in D_2_O and adjusted to 50 mg mL^-1^. To precisely test the absorbance of OH^-^ in the crystal, the UCNPs solutions (in D_2_O) were separated in triplicate, the content of OH^-^ were determined from its absorbance near 3400 cm^-1^, measured with FTIR for three times.

According to Beer-Lambert law, absorbance of OH^-^ (A_OH_), is:

A_OH_ =ε*L*C_OH_

where *ε* is the absorption coefficient, *L* is the cell path length, and *C_OH_* is concentrations of NaOH, the *ε* and *L* were constant with low OH^-^ contents and same measurement conditions.

The NaOH solutions (in D_2_O) was used as the standard reagent (**Figure S10)**, thus:

C_OH_ = A_OH_/4.12 mol L^-1^

The concentration of UCNPs is:

C_UCNPs_ = 50 mg mL^-1^/M_UCNPs_

Where *M_UCNPs_* is 206 g mol^-1^ for the co-doped (Yb/Er) UCNPs and 170 g mol^-1^ for the single doped (2% Er) UCNPs.

Thus, the OH^-^ content (*x*) thus is:

*x* = C_OH_/C_UCNPs_

According to the three parallel measurements, the OH^-^ contents (*x*) in the bare core crystal lattice with samples (Dry, OH1-4) were obtained, as listed in **Table S2**.

However, it should be notice that for the core/shell structures, there may a technical limitation to determine the OH^-^ content directly. Because we find that (i) the larger the particle size is, the smaller the relevant saturation concentration in D_2_O will be. Specifically, the core/shell-structured nanoparticles could only be uniformly dispersed without precipitation at a concentration less than 20 mg mL^-1^, instead of 50 mg mL^-1^ for bare core, and (ii) for the core/shell structure, the volume of the core area where the OH^-^ impurities locate only takes a small share of the entire particle volume. Therefore, nanostructures with low OH^-^ contents suffer from the dilemma that the signal intensity as well as the signal-to-noise ratio (S/N) of the FTIR measurements may not be strong enough for detection. However, we measured the OH^-^ contents in dry (lower limit) and OH4 (upper limit) core/shell structures. As listed in the **Table** **S3**, we find that (i) the dry shell coating will not introduce additional OH^-^ contents into the system (testified by dry core@dry shell sample), and (ii) for the core/shell OH4 sample, despite the error bar is relatively large, the calculated *x* value (0.129±0.025) is well in line with the result in bare core OH4 structure (0.120±0.008).

Besides, in theory, we also have reasons that coating a dry shell will not change the OH^-^ content in the inner core remarkably. Because: (i) there is no significant difference between the synthesis conditions for shell coating (300 ^o^C, 90 min) and nucleation (300 ^o^C, 60 min). Therefore, the core area should be considered to be thermodynamically stable during the dry shell coating process. In other words, if the OH^-^ group could not be removed during the nucleation, it will be naturally reserved in shell coating process. (ii) Previous works have also admitted that it is almost impossible that the element component of a core undergoes a significant variation via traditional shell coating strategy. Although elements diffusion may occur at the core-shell interface, the influence is proved to be relatively weak, and not enough to shake the credibility of our conclusions.^S2^

In that cases, in this work, our analysis based on a conclusion that the OH^-^ contents will essentially unchanged before and after the dry shell coating.

**Simulation model**

(1) **Setting up the parameters in simulation**

The Monte Carlo simulation model of UC was upgraded from our previous work,^S3^ which based on the hypotheses of three-dimensional random wandering of the excited states in the sublattice (composed by sensitizer and activator ions), and UC events resulting from “collision” of two or more excited states. In this work, the energy-loss pathways with OH^-^ was added in the simulation that led to higher non-radiative recombination rate and lower UC efficiencies, depending on the OH^-^ content. The relevant simulation parameters are listed in **Table S4**. Among these previous parameters, the de-excitation/recombination rate of sensitizer (*S1* energy level) is the only one that has been modified. According to the measured experiment results (decay lifetime of Yb^3+^ excited state in the Dry UCNPs, *τ*=1.4 ms, as shown in **Figure S11**), it has been slightly modified from 1000 s^-1^ to 735 s^-1^ (R= 1/τ).

(2) **Modelling the simulation**

To deal with the OH^-^ effects, the sensitizer (Yb^3+^)/activator (Er^3+^) are simplified to two/ three energy levels system, respectively (labeled as *S_0_,_1_* and *A_0,1,2_*_,_ respectively).

In this case, the microscopic picture of UC luminescence in the 20%Yb, 2%Er system can be described as follows: two *S_1_* excited states transfer their energy after different migration paths to one ground state activator in a fix order (firstly, from one *S_1_* to *A_0_*, after that, from another *S_1_* to *A_1_*) to populate the *A_2_* energy level, resulting in one UC emission photon. Following this process, the negative effects of OH^-^ should include the quenching of the *S_1_* states of the two sensitizers, *A_1_ and A_2_* state, *i.e.* the overall quenching factor Γ is expressed as**:**

Γ=Γ_Er_*(Γ_Yb_)^2^=Γ_A1_*Γ_A2_*(Γ_S1_)^2^

The parameter Γ_Yb_ (*i.e.*, Γ_S1_) is discussed in the following section (calculated from the decay lifetimes of OH^-^ involved Yb^3+^ ions).

In single dopant case like NaYF_4-_*_x_*(OH)*_x_*:2%Er@NaYF_4_ system, however, the situation is different. Under 980 nm excitation, Er^3+^ ions play the dual roles of sensitizer and activator. Dominant UC process can be regarded as “collision” of two Er^3+^ ions of *A_1_* state. The quenching factor Γ_Er_’ can thus be expressed as**:**

Γ_Er_’ = (Γ_A1_)^2^*Γ_A2_

From the above two expressions it is obvious that the quenching factor of single Er dopant case (*i.e.,* Γ_Er_’) is not equal to the Γ_Er_ in Er/Yb co-dopant system (*i.e.,* Γ_A1_*Γ_A2_). In order to obtain an analytical expression of Γ capable to compare with experimental data, approximation is needed.

Approximation 1, where Γ is calculated as Γ_Er_’*(Γ_Yb_)^2^, which means that the quenching in the single dopant case is regarded as the quenching of the activator in co-dopant case. The shortage of this approximation is that the quenching process on *A_1_* state will be overestimated (count Γ_A1_ two times).

Approximation 2, where Γ is calculated by Γ_Er_’*Γ_Yb_. In this case, the parameter (Γ_S1_)^2^ is approximated to Γ_S1_*Γ_A1_ (*i.e.*, using one Er→Er energy transfer process to replace one Yb→Er energy transfer process).

Since both approximations have certain limit, we have compared the simulations based on the approximations with the experimental data. As shown in **Figure S14**, the Approximation 2 describes better the OH^-^ dependence of the UCL. Therefore, we have chosen the Approximation 2 in this case.

(3) Determination of parameters Γ_Er_’, Γ_Yb_ and *R_Yb-OH_*

From the UCL spectra and FTIR spectra of only Er^3+^ doped UCNPs (**Figure 2a** and **Figure S9**), the parameter Γ_Er_’ can be obtained. Taking OH^-^ content (*x*) as the sole fitting parameter for the UCL intensity (*y*) of Er^3+^, an exponential relation was obtained for single doped UCNPs (**Figure 2a inset**):

Γ_Er_’ = e^-^*^x^*^/^*^c1^*

where *c* was determined to be 0.072 from fitting, and *x* is the OH^-^ content in the NaYF_4-_*_x_*(OH)*_x_*:2%Er@NaYF_4_ nanoparticles.

For the Yb-OH interaction, in theory, the OH^-^ influenced Yb^3+^ dynamics (^2^F_5/2_→^2^F_7/2_ transition) can be described by a so-called “energy hopping” mode. According to Zusman and Burshtein,^S4, S5^ energy hoping between donors leads to the following expressing for its luminescence dynamics:

$$I(t)=I(0)exp\left\{ -\frac{t}{\tau_{D}}-\gamma\sqrt{t} \right\}exp\left( -\omega t \right)$$

where $\omega$is the an ensemble-averaged migration-assisted energy-transfer rate between donor (Yb^3+^) and acceptor (OH^-^),*τ_D_* is the intrinsic lifetime of the donor, $\gamma$is a parameter related to acceptor concentration and microscopic donor-acceptor interact rate. Obviously, it is difficult to get all these parameters from experiment.

In that case, we try to reveal the Yb^3+^-OH interaction from simulation. We calculate the OH^-^ influenced Yb^3+^→Yb^3+^ energy migration dynamics in an infinite NaYF_4_:20%Yb sub-lattice. Typically, (1) OH^-^ group was randomly brought into Yb^3+^ sub-lattice with its content varying from 0 to 0.120; (2) to simplify the model, it was assumed that each OH^-^ group interacted directly only with one nearest Yb^3+^ ion, the influence of OH^-^ on other Yb^3+^ ions was realized indirectly through the bridge of Yb^3+^→Yb^3+^ energy migration process; (3) the negative effect of OH^-^ group was to introduce an additional non-radiative rate to its nearest Yb^3+^ ion. We defined the parameter *R_Yb-OH_* as the recombination rate of Yb^3+^ excited states with an adjacent OH^-^ quencher. On the contrary, the parameter *R_Yb_* was a constant which was the recombination rate of Yb^3+^ ions without OH^-^ in the close vicinity (calculated to 735 s^-1^, from the decay lifetime of Yb^3+^ in the Dry UCNPs, as shown in **Figure S11**).

According to our simulation, Yb^3+^ decay lifetime was found to descend steeply with the increase of *R_Yb-OH_*. Interestingly, it will follow a quasi-exponential form when *R_Yb-OH_* is relatively small (**Figure S12**, exponential relationship should be performed as a straight line in this semi-logarithmic figure, where *R_Yb-OH_* is varied from 1000 to 50000 s^-1^). The possible reason behind might be the huge difference between *R_Yb-Yb_* (Yb^3+^→Yb^3+^ energy migration rate, set as 10^5^ s^-1^ from our previous work^S3^) and *R_Yb-OH_* (set as 2100 s^-1^, according to our simulation, see below). In that case, two types of Yb^3+^ ions (with and without OH^-^ in the close vicinity) are connected efficiently via Yb^3+^→Yb^3+^ energy migration, resulting in a quasi-uniform quenching effect. However, if *R_Yb-OH_* is larger than 5000 s^-1^, the quasi-exponential relationship will be broken (**Figure S12**).

Fortunately, in our system, *R_Yb-OH_* falls in that range (optimized to 2100 s^-1^ from simulation, see below). Therefore, in our system (NaYF_4-_*_x_*(OH)*_x_*:20%Yb, 2% Er@NaYF_4_ core-shell nanostructure), the internal OH^-^ induced quenching for Yb^3+^ can be expressed as:

Γ_Yb_ = e^-^*^x/c2^*

where *x* is the internal OH^-^ content of the nanoparticle, and constant *c_2_* was determined to be 0.119 from fitting. It should be noticed that the exponential relationship form of *Γ_Yb_* is also directly confirmed by our experimental data (**Figure 2b**).

Based on Approximation 2 strategy, the total quenching efficiency of the co-doped UCNPs is a product of the impact of Γ_Er_’and Γ_Yb_, *i.e.,*

Γ_Yb/Er_ = Γ_Er_ * (Γ_Yb_)^2^≈Γ_Er_’ * Γ_Yb_

In additional, the key parameter, *i.e., R_Yb-OH_*, is obtained from the comparison of simulation and experiment results. We varied *R_Yb-OH_* from 1000 to 5000 s^-1^ during the luminescence dynamics fitting. The simulated steady-state UC emission intensity results (under the 100 W cm^-2^ 980 nm excitation) are provided in **Table S5** (1000 s^-1^), **Table S6** (1500 s^-1^), **Table S7** (2100 s^-1^), **Table S8** (2500 s^-1^), **Table S9** (3000 s^-1^) and **Table S10** (5000 s^-1^), respectively. These simulation results are pooled in **Figure S5b**. Besides, the internal OH^-^ influenced decay lifetimes of Yb^3+^ ions are also simulated (as shown in **Figure S5a**). We find these experimental data (steady-state UC emission intensity and Yb^3+^ decay lifetime) could be perfectly reproduced when the migration rate is setting to 2100 s^-1^ (**Figure 2c** and **S5**).


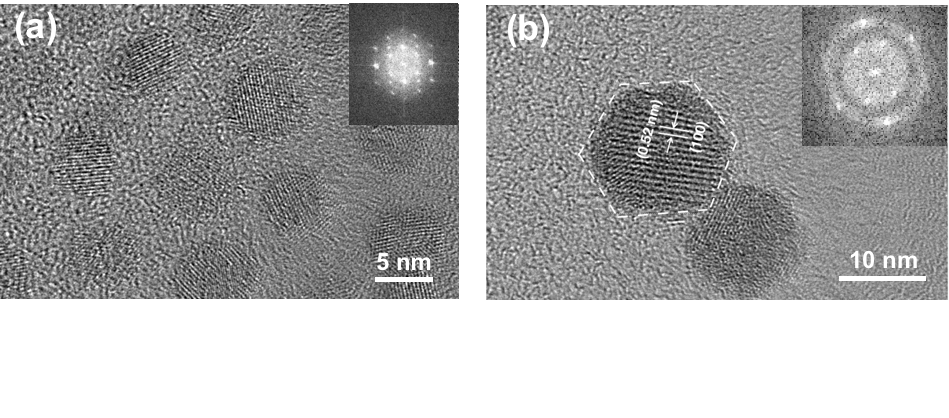


**Figure S1**. HRTEM image and Fourier-transform diffraction patterns (upper right) of (a) core UCNPs and (b) core-shell Dry UCNPs.


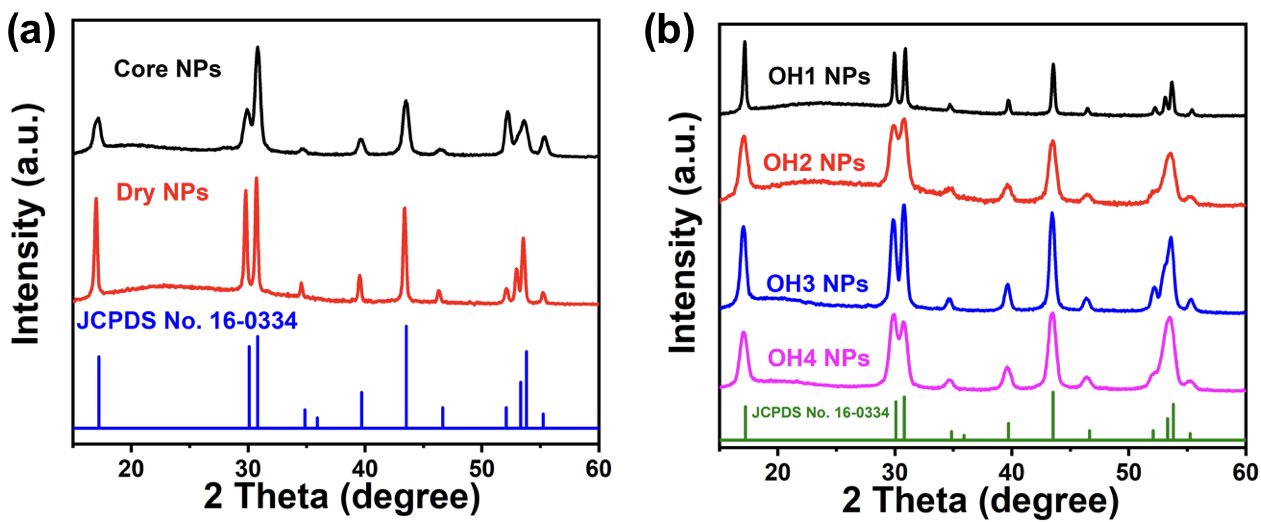


**Figure S2**. XRD patterns of (a) core and Dry core-shell UCNPs; (b) core–shell UCNPs with different content OH^-^ (OH1, OH2, OH3 or OH4). JCPDS No. 16-0334: the standard diffraction pattern of hexagonal phase.


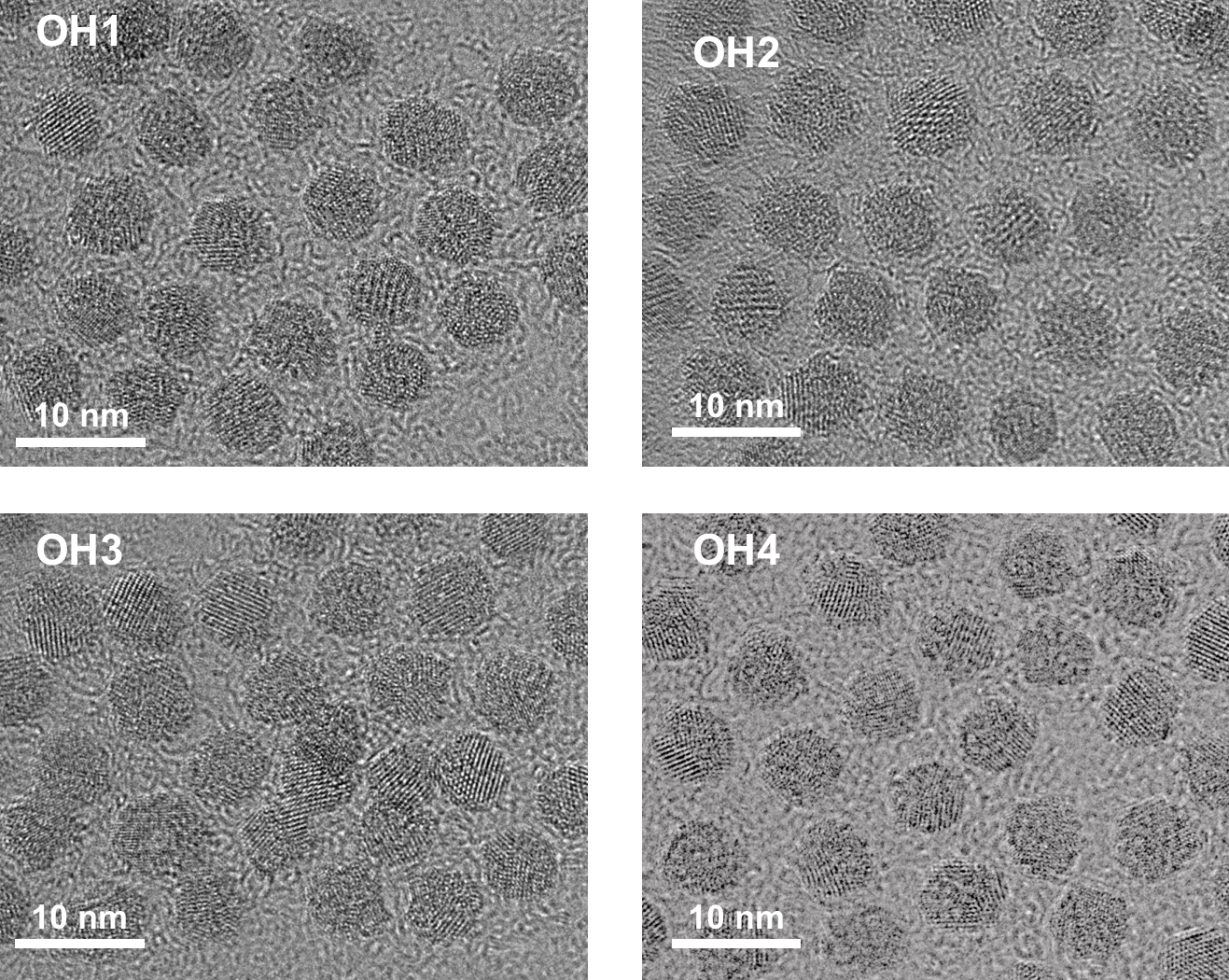


**Figure S3.** Typical TEM images of NaYF_4-x_(OH)_x_:Yb,Er bare core UCNPs (OH1-4, diameters all around 7.5 nm).


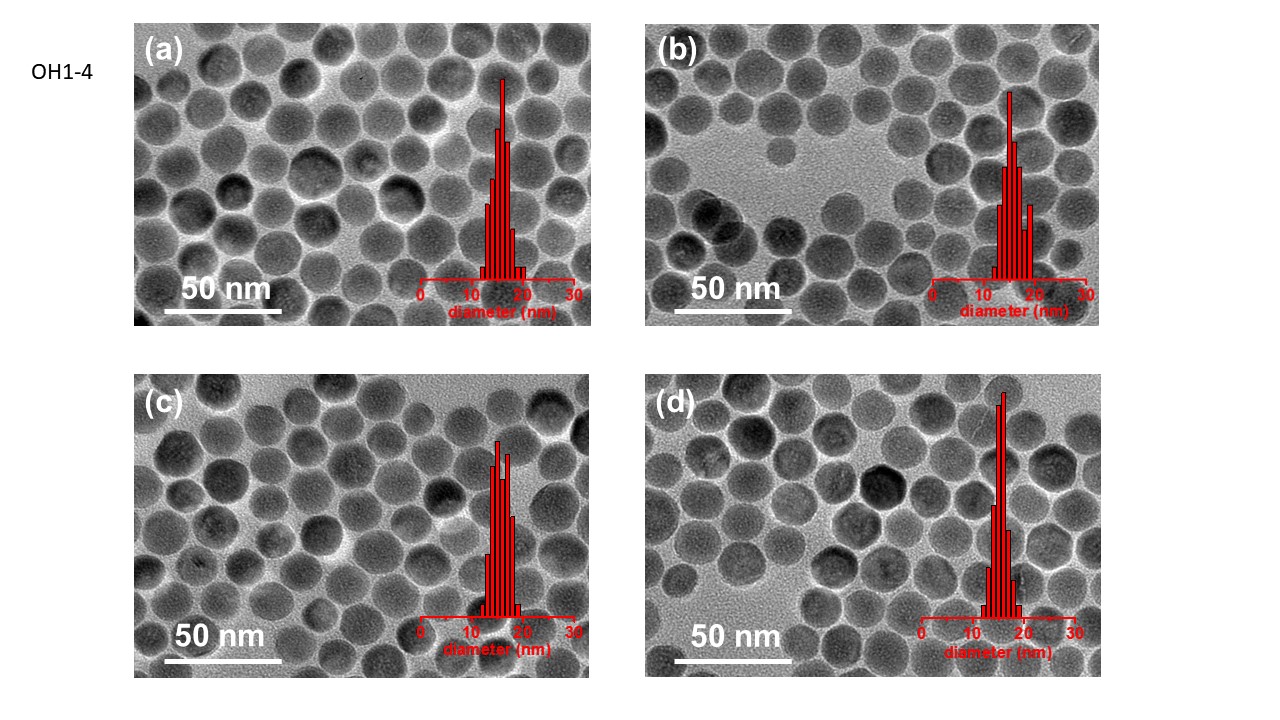


**Figure S4**. Typical TEM images of (a) OH1, (b) OH2, (c) OH3 and (d) OH4 Core@Shell UCNPs (diameters all around 15 nm).


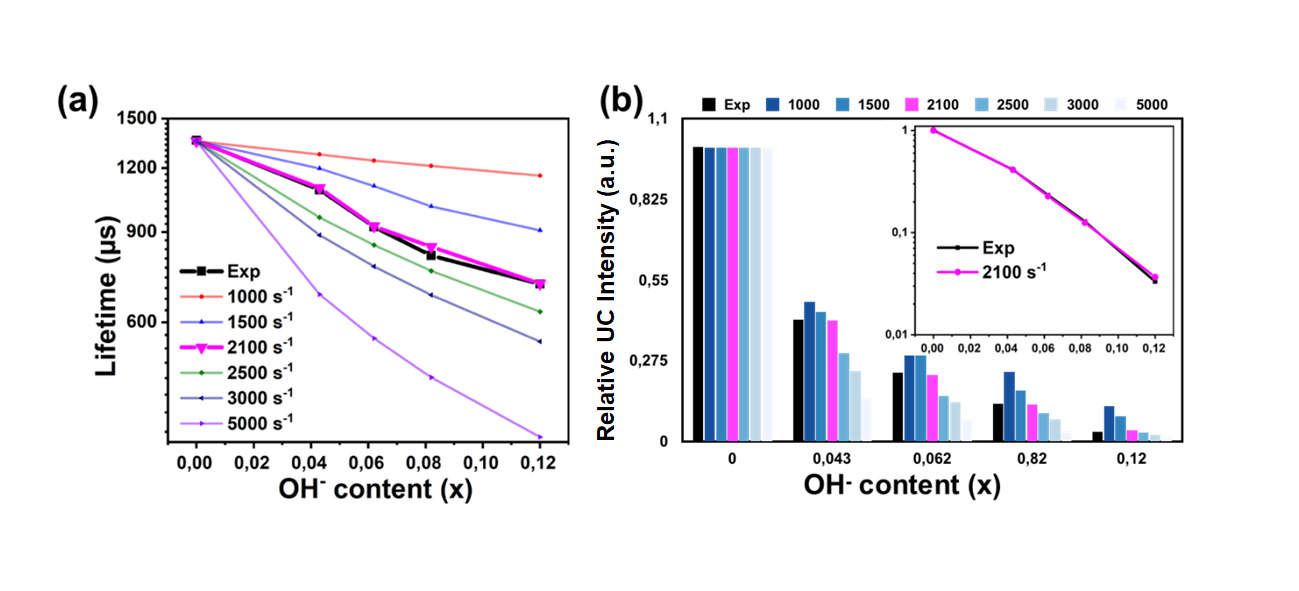


**Figure S5**. (a) Experimental and simulated OH^-^ content dependence of Yb^3+^ emission decay constant of co-doped UCNPs (NaYF_4-_*_x_*(OH)*_x_*:Yb,Er@NaYF_4_). Experimental results (Exp, black line) and simulation results of different recombination rates (*R_Yb-OH_*: 1000 s^-1^ - 5000 s^-1^). (b) Relative green and red UCL integrated intensity of co-doped core-shell UCNPs (NaYF_4-_*_x_*(OH)*_x_*:Yb,Er@NaYF_4_) versus OH^-^ content. Black lines are the experimental results, lines in other colors are the simulation results with different recombination rates of Yb^3+^ (*R_Yb-OH_*: 1000 s^-1^ - 5000 s^-1^, indicated in the figure). All these simulation results are normalized by the OH^-^ free sample. It is worth to notice that **Figure S5a** is in semi-logarithmic scale. Y-axis is in Ln scale, and x-axis is in linear scale. Therefore, the exponential relationship in Eq.3 (*i.e.,* Γ_Yb_ = e^-^*^x^*^/c2^) should be performed as a straight line in **Figure S5a**, which is consistent with our simulation results (while the *R_Yb-OH_* is tuned from 1000 s^-1^ to 2500 s^-1^).


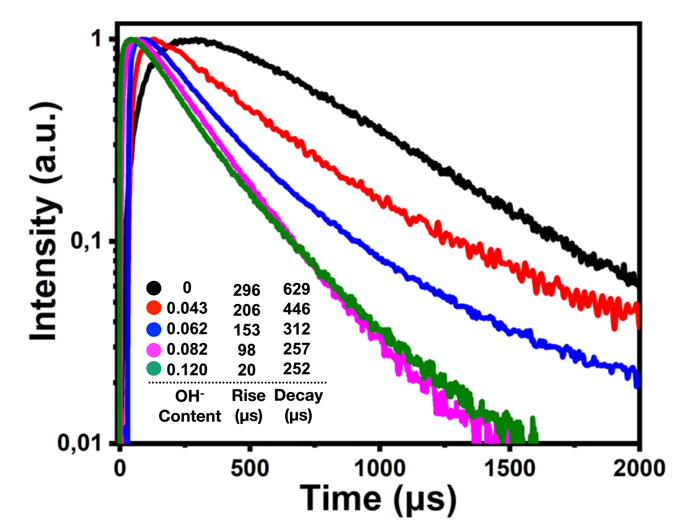


**Figure S6**. (a) OH^-^ content dependence of full time trace UC red emission of co-doped core-shell UCNPs (NaYF_4-_*_x_*(OH)*_x_*:Yb,Er@NaYF_4_) monitored at ~ 654 nm.


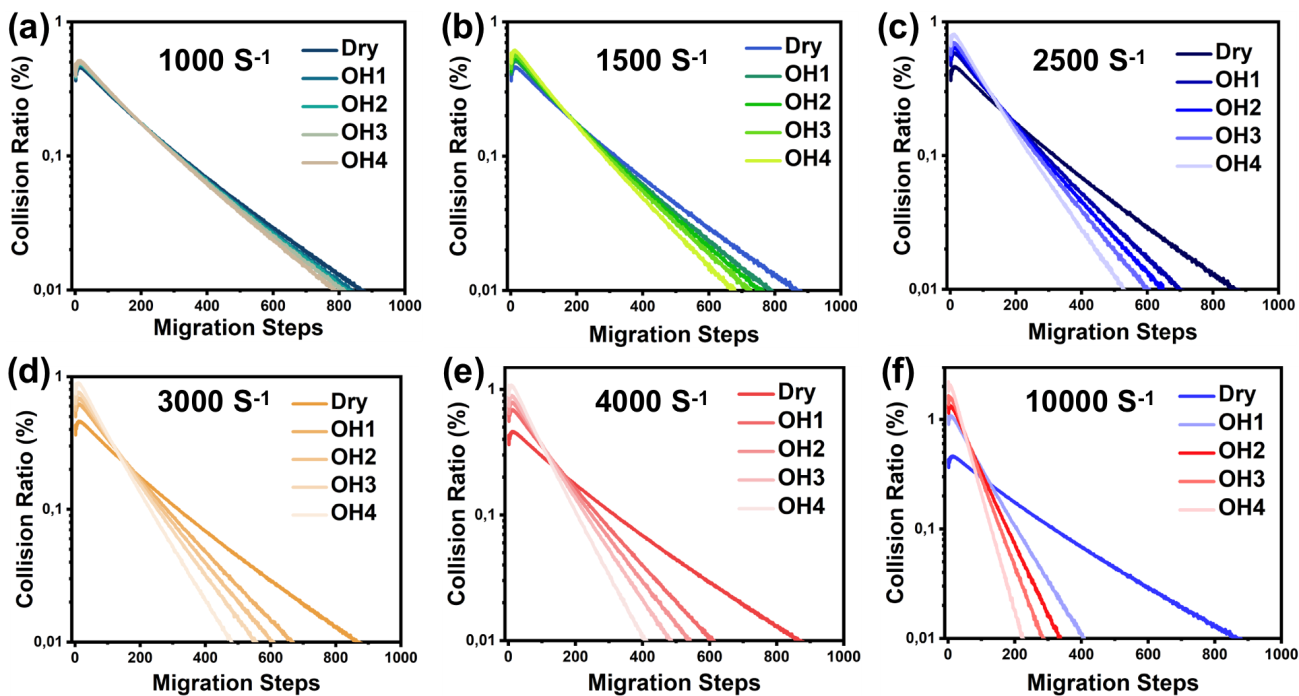


**Figure S7**. The simulated decay curves of Yb^3+^ in Yb^3+^/Er^3+^@Y core-shell UCNPs with different content of OH^-^. Recombination rate of OH^-^ influenced Yb^3+^ varied from 1000 s^-1^ to 10000 s^-1^.


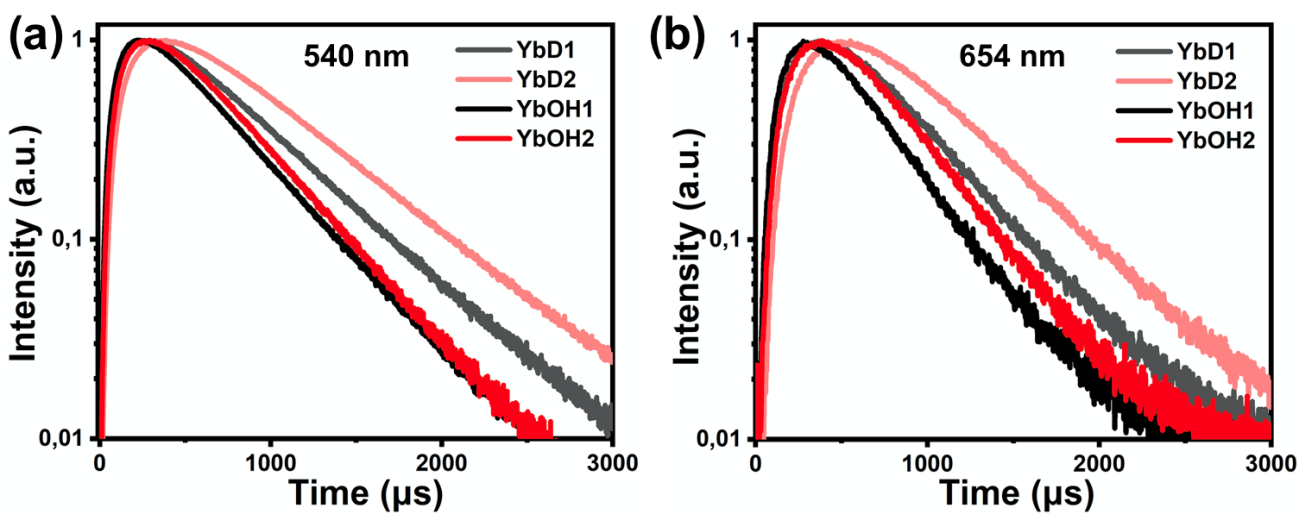


**Figure S8**. The UCL green (**a**, 540 nm) and red (**b**, 654 nm) traces of the nanoparticles Yb,Er/Yb(OH)*_x_*/Nd with different middle layer samples (*i.e.* thickness and OH^-^ content) under 800 nm excitation. The thickness is ca. 2.3 nm for YbD1 and YbOH1, and 3.3 nm for YbD2 and YbOH2. YbD1 and YbD2 are samples without internal OH^-^, YbOH1 and YbOH2 are samples with internal OH^-^ in the middle layer.


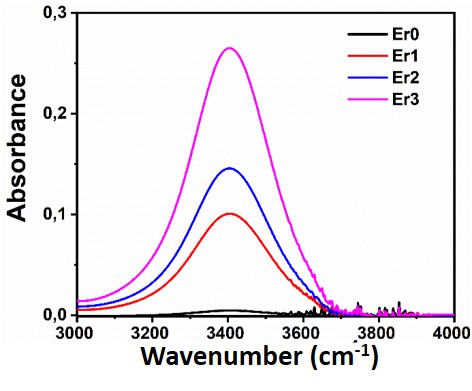


**Figure S9**. The FTIR spectra of Er^3+^ singly doped NPs (NaYF_4-_*_x_*(OH)*_x_*: 2%Er @NaYF_4_) with different internal OH^-^ contents (samples were dispersed in D_2_O solvent).


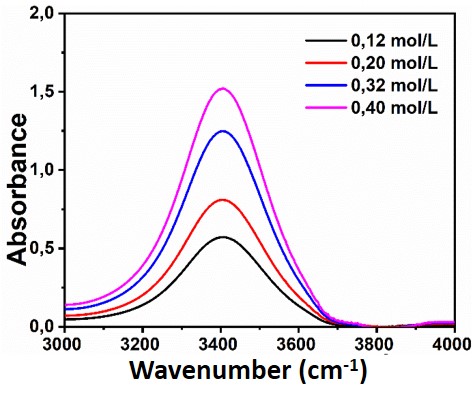


**Figure S10**. The FTIR spectra of NaOH standard (with different concentration in D_2_O solvent).


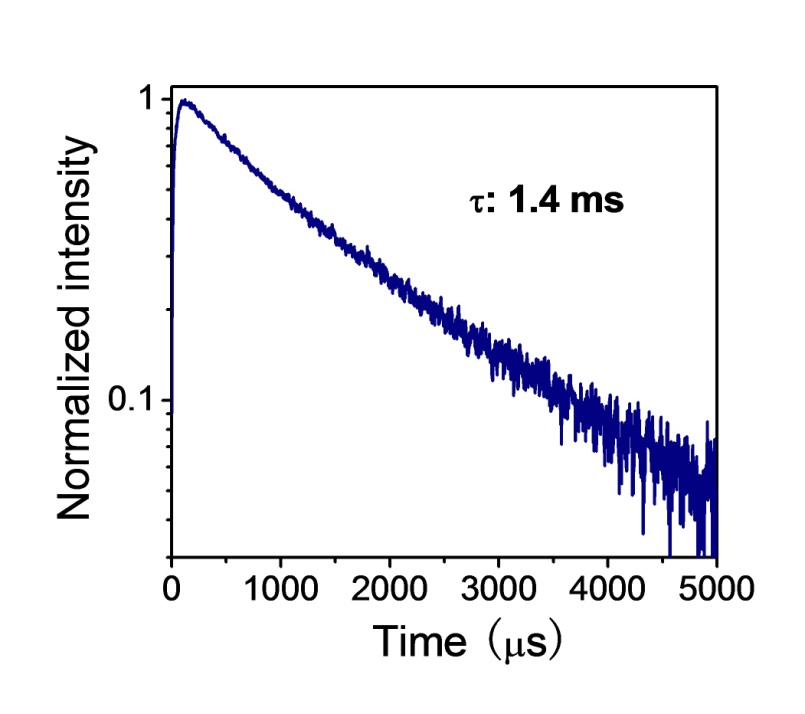


**Figure S11**. The measured time trace of Yb^3+^ in the Dry-UCNPs (Ex: 980 nm, Em: 1020 nm).


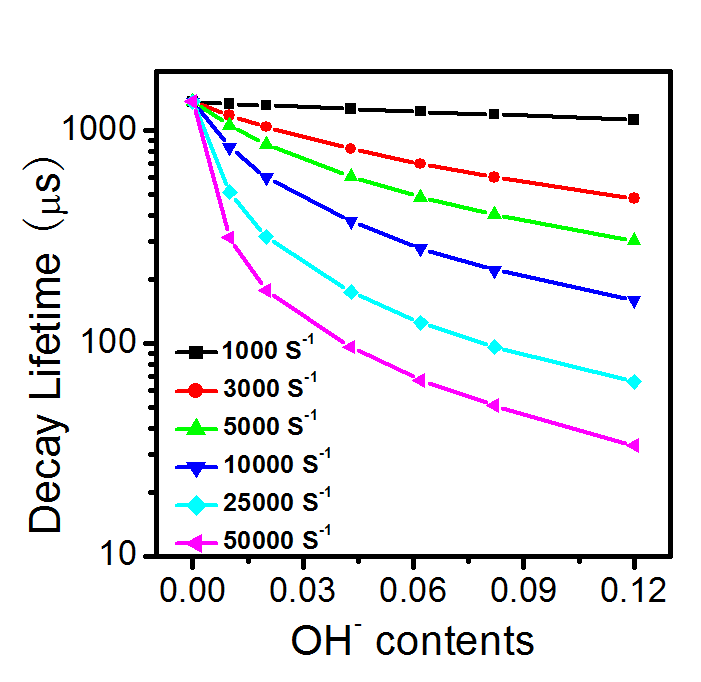


**Figure S12**. Simulated results of internal OH^-^ content dependent of Yb^3+^ decay lifetimes (with different *R_Yb-OH_* values). Exponential relationship should be performed as a straight line in this semi-logarithmic figure, which fits the situations of relatively weak *R_Yb-OH_* values (less than 3000 s^-1^).


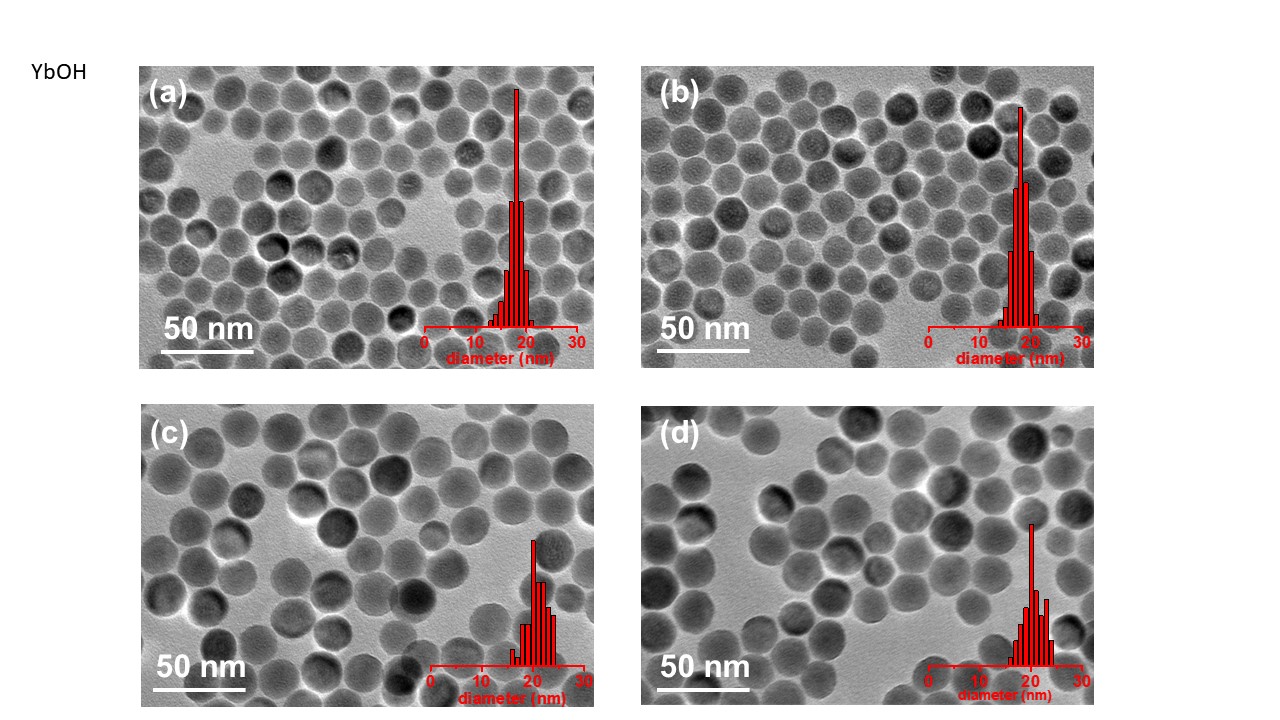


**Figure S13**. Typical TEM images of (a) YbD1 (~ 17 nm), (b) YbOH1 (~ 17 nm), (c) YbD2 (~ 20 nm) and (d) YbOH2 (~ 20 nm) nanoparticles.


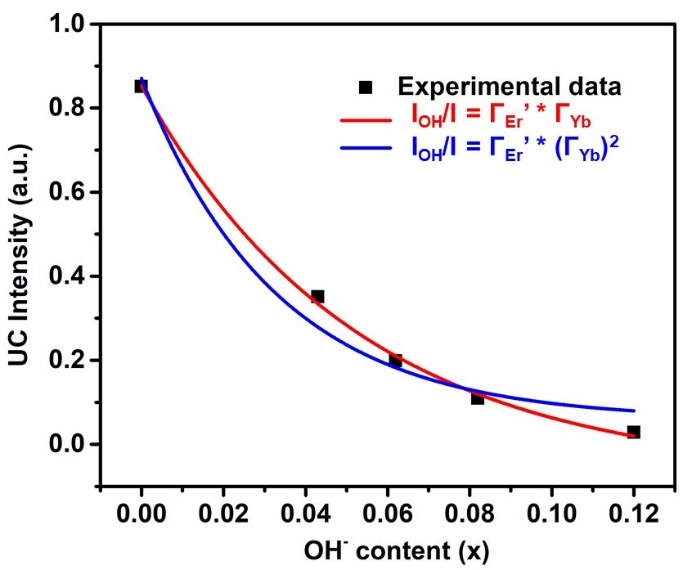


**Figure S14**. Internal OH^-^ content related UCL intensity (integrated from 500 to 700 nm) of NaYF_4-_*_x_* (OH)*_x_*:Yb/Er@NaYF_4_ nanoparticles. Black dots are the experimental results, the blue/red curves are the simulation results from Approximation 1 and 2, respectively (part of the data are already been presented in Figure 2c).

**Table S1**. The measured UC quantum yields (Φ_UC_) of the series NaYF_4-x_(OH)_x_:Yb,Er@NaYF_4_ nanoparticles (x varied from 0 to 0.12). The testing method is the repeat of the previous work (*ρ*=5.0 W cm^-2^, T = 293 K, some data are too weak to give a reliable result).^S6^ *

| **Samples** | Φ_UC, 523_ | Φ_UC, 544_ | Φ_UC, 655_ | Φ_UC, 806_ | Φ_UC, 826_ | Φ_UC, total_ (%) |
| --- | --- | --- | --- | --- | --- | --- |
| **Dry** | 3(1)* 10^-5^ | 2(1) * 10^-4^ | 8(2) * 10^-3^ | 2.3(0.6) * 10^-3^ | 2.4(0.7) * 10^-3^ | 1.3(0.2) |
| **OH1** | 1* 10^-5^ | 7(2) * 10^-5^ | 2(1) * 10^-3^ | 8(2) * 10^-4^ | 9(3) * 10^-4^ | 0.4(0.1) |
| **OH2** | - | 3(1) * 10^-5^ | 9(3) * 10^-4^ | 5(2) * 10^-4^ | 4(2) * 10^-4^ | 0.2(0.1) |
| **OH3** | - | - | - | - | - | - |
| **OH4** | - | - | - | - | - | - |

*For UCL quantum yield, it should be noted that its value is always not a precise parameter since it is excitation power density dependent. Therefore, it is difficult to obtain an objective and credible value since excitation beam profile and collection configuration may differ from setup to setup. Even in the same integrating sphere setup, the non-uniformed beam profile means the QY value is in fact a collection of QYs subjected to different power excitations. However, QY values are qualitatively accurate, and reliable for the comparison propose.

**Table S2**: The measured OH^-^ contents (*x*) in the series core samples (Dry, OH1-4).

| **Samples** | OH^-^ contents (*x*) | | | Error |
| --- | --- | --- | --- | --- |
| **Dry** | ‹0.01 | ‹0.01 | ‹0.01 | -- |
| **OH1** | 0.043 | 0.038 | 0.045 | 12% |
| **OH2** | 0.062 | 0.054 | 0.065 | 13% |
| **OH3** | 0.082 | 0.076 | 0.085 | 7% |
| **OH4** | 0.120 | 0.116 | 0.128 | 7% |

Error bias is calculated by (*x*’-*x*)/*x*

**Table S3**: The measured OH^-^ contents (*x*) in the NaYF_4-x_(OH)_x_@NaYF_4_ core/shell structures (Dry and OH4).

| **Samples** | **OH^-^ contents (*x* value)** | | | **Error** | |
| --- | --- | --- | --- | --- | --- |
| **Dry** | <0.01 | <0.01 | <0.01 | | - |
| **OH4** | 0.104 | 0.135 | 0.147 | | 19% |

Error bias is calculated by (*x*’-*x*)/*x*

**Table S4**. The simulation parameters used in this work (*S_0,1_* and *A_0,1,2_* are the energy levels of sensitizer Yb^3+^ and activator Er^3+^ respectively). It should be noticed that the selecting of most of the parameters can be traced back to our previous work.^S3^

| **Parameters** | Value |
| --- | --- |
| \| Simulation time period (s) \| \| --- \| \| Time step (s) \| \| Recombination rate of *S_1_* (s^-1^)  Recombination rate of *S_1_* with OH^-^ (s^-1^) \| \| Recombination rate of *A_1_* (s^-1^) \| \| Recombination rate of *A_2_* (s^-1^) \| \| Energy migration rate of *S_1_→S_0_* (s^-1^) \| \| Energy transfer rate of *S_1_→A_0_* (s^-1^) \| \| Energy transfer rate of *S_1_→A_1_* (s^-1^) \| \| Energy transfer rate of *A_1_→S_0_* (s^-1^) \| \| Energy migration rate of *A_1_→A_0_* (s^-1^) \| \| Energy transfer rate of *A_1_→A_1_* (s^-1^) \| \| Absorption cross section of *S_0_* (cm^2^) \| \| Absorption cross section of *A_0_* (cm^2^) \| \| Surface quenching rate (s^-1^) \| \| Quantum yield of *A_2_* state \| | \| 3 \| \| --- \| \| 10^-6^ \| \| 735  1000 -- 5000 \| \| 10^3^ \| \| 7∙10^3^ \| \| 10^5^ \| \| 2.5∙10^4^ \| \| 3.2∙10^3^ \| \| 1.0∙10^4^ \| \| 5∙10^3^ \| \| 600 \| \| 1.17∙10^-20^ \| \| 1.7∙10^-21^ \| \| 10^5^ \| \| 50% \| |

**Table S5**. Simulated UC efficiency for Yb^3+^/Er^3+^ co-doped UCNPs with different contents of OH^-^ (*R_Yb_* is 735 s^-1^, *R_Yb-OH_* is 1000 s^-1^).

| **OH^-^ content** | 0 | 0.043 | 0.062 | 0.082 | 0.120 |
| --- | --- | --- | --- | --- | --- |
| **Absorbed photons** | 268029 | 267995 | 268008 | 268046 | 268001 |
| **Recombined on the S1 state** | 78.32% | 78.02% | 78.67% | 79.28% | 80.34% |
| **Recombined on the A1 state** | 18.73% | 17.53% | 17.09% | 16.75% | 16.03% |
| **Recombined on the A2 state** | 2.48% | 2.22% | 2.12% | 1.99% | 1.81% |
| **UC emission photons** | 3321 | 2974 | 2895 | 2652 | 1027 |
| **UC efficiency** | 1.24% | 1.11% | 1.08% | 0.99% | 0.90% |

**Table S6**. Simulated UC efficiency for Yb^3+^/Er^3+^ co-doped UCNPs with different contents of OH^-^ (*R_Yb_* is 735 s^-1^, *R_Yb-OH_* is 1500 s^-1^).

| **OH^-^ content** | 0 | 0.043 | 0.062 | 0.082 | 0.120 |
| --- | --- | --- | --- | --- | --- |
| **Absorbed photons** | 268029 | 268091 | 268034 | 267996 | 267993 |
| **Recombined on the S1 state** | 78.32% | 80.48% | 81.86% | 83.07% | 84.95% |
| **Recombined on the A1 state** | 18.73% | 15.94% | 14.93% | 14.16% | 12.78% |
| **Recombined on the A2 state** | 2.48% | 1.79% | 1.61% | 1.38% | 1.13% |
| **UC emission photons** | 3321 | 2381 | 2145 | 1883 | 1515 |
| **UC efficiency** | 1.24% | 0.89% | 0.80% | 0.68% | 0.57% |

**Table S7**. Simulated UC efficiency for Yb^3+^/Er^3+^ co-doped UCNPs with different content of OH^-^ (*R_Yb_* is 735 s^-1^, *R_Yb-OH_* is 2100 s^-1^)..

| **OH^-^ content** | 0 | 0.043 | 0.062 | 0.082 | 0.120 |
| --- | --- | --- | --- | --- | --- |
| **Absorbed photons** | 268029 | 267984 | 267980 | 267976 | 267953 |
| **Recombined on the S1 state** | 78.32% | 82.70% | 84.47% | 86.14% | 88.29% |
| **Recombined on the A1 state** | 18.73% | 14.45% | 13.12% | 11.90% | 10.24% |
| **Recombined on the A2 state** | 2.48% | 1.42% | 1.20% | 0.98% | 0.73% |
| **UC emission photons** | 3321 | 1946 | 1641 | 1295 | 933 |
| **UC efficiency** | 1.24% | 0.73% | 0.61% | 0.48% | 0.35% |

**Table S8**. Simulated UC efficiency for Yb^3+^/Er^3+^ co-doped UCNPs with different content of OH^-^ (*R_Yb_* is 735 s^-1^, *R_Yb-OH_* is 2500 s^-1^)..

| **OH^-^ content** | 0 | 0.043 | 0.062 | 0.082 | 0.120 |
| --- | --- | --- | --- | --- | --- |
| **Absorbed photons** | 268029 | 268007 | 268024 | 267999 | 268038 |
| **Recombined on the S1 state** | 78.32% | 83.88% | 85.96% | 87.53% | 89.84% |
| **Recombined on the A1 state** | 18.73% | 13.58% | 11.99% | 10.79% | 9.03% |
| **Recombined on the A2 state** | 2.48% | 1.27% | 1.02% | 0.84% | 0.57% |
| **UC emission photons** | 3321 | 1700 | 1296 | 1082 | 762 |
| **UC efficiency** | 1.24% | 0.63% | 0.48% | 0.40% | 0.28% |

**Table S9**. Simulated UC efficiency for Yb^3+^/Er^3+^ co-doped UCNPs with different content of OH^-^ (*R_Yb_* is 735 s^-1^, *R_Yb-OH_* is 3000 s^-1^)..

| **OH^-^ content** | 0 | 0.043 | 0.062 | 0.082 | 0.120 |
| --- | --- | --- | --- | --- | --- |
| **Absorbed photons** | 268029 | 267941 | 268066 | 268031 | 267985 |
| **Recombined on the S1 state** | 78.32% | 85.32% | 87.38% | 89.09% | 91.20% |
| **Recombined on the A1 state** | 18.73% | 12.45% | 10.91% | 9.60% | 7.91% |
| **Recombined on the A2 state** | 2.48% | 1.12% | 0.85% | 0.66% | 0.44% |
| **UC emission photons** | 3321 | 1505 | 1088 | 843 | 617 |
| **UC efficiency** | 1.24% | 0.56% | 0.41% | 0.31% | 0.23% |

**Table S10**. Simulated UC efficiency for Yb^3+^/Er^3+^ co-doped UCNPs with different content of OH^-^ (*R_Yb_* is 735 s^-1^, *R_Yb-OH_* is 5000 s^-1^).

| **OH^-^ content** | 0 | 0.043 | 0.062 | 0.082 | 0.120 |
| --- | --- | --- | --- | --- | --- |
| **Absorbed photons** | 268029 | 268084 | 268023 | 268045 | 268045 |
| **Recombined on the S1 state** | 78.32% | 88.97% | 91.16% | 92.66% | 94.38% |
| **Recombined on the A1 state** | 18.73% | 9.69% | 7.96% | 6.73% | 5.24% |
| **Recombined on the A2 state** | 2.48% | 0.67% | 0.44% | 031% | 0.19% |
| **UC emission photons** | 3321 | 899 | 621 | 414 | 268 |
| **UC efficiency** | 1.24% | 0.34% | 0.23% | 0.15% | 0.10% |

**References:**

S1. Alain Baumer, M. G., Wilfrid E. Klee, Determination of OH ions in Hydroxyfluorapatites by Infrared Spectroscopy. *Bulletin de Minéralogie* **108**, 145-152 (1985).

S2. Würth, C., Fischer, S., Grauel, B., Alivisatos, A P., Resch-Genger, U., Quantum Yields, Surface Quenching, and Passivation Efficiency for Ultrasmall Core/Shell Upconverting Nanoparticles. *Journal of the American Chemical Society* **140**, 4922 – 4928 (2018).

S3. Zuo, J. *et al.* Precisely Tailoring Upconversion Dynamics via Energy Migration in Core-Shell Nanostructures. *Angewandte Chemie International Edition* **57**, 3054-3058 (2018).

S4. Zusman, L. D., Kinetics of Luminescence Damping in the Hopping Mechanism of Quenching. *Soviet Physics—JETP* **46**, 347-351 (1977).

S5. Burshtein, A. I., Hopping Mechanism of Energy Transfer. *Soviet Physics—JETP* **35**, 882-885 (1972).

S6. Meijer, M. S. *et al.* Absolute Upconversion Quantum Yields of Blue-Emitting LiYF_4_:Yb^3+^,Tm^3+^ Upconverting Nanoparticles. *Physical Chemistry Chemical Physics* **20**, 22556-22562 (2018).
